# Robust Wnt signaling is maintained by a Wg protein gradient and Fz2 receptor activity in the developing *Drosophila* wing

**DOI:** 10.1242/dev.174789

**Published:** 2019-08-09

**Authors:** Varun Chaudhary, Swapnil Hingole, Jana Frei, Fillip Port, David Strutt, Michael Boutros

**Affiliations:** 1 German Cancer Research Center (DKFZ), Division Signaling and Functional Genomics and Department of Cell and Molecular Biology, Medical Faculty Mannheim, Heidelberg University, Im Neuenheimer Feld 580, 69120 Heidelberg, Germany; 2 Department of Biological Science, Indian Institute of Science Education and Research Bhopal, Bhopal Bypass Road, Bhauri, Bhopal 462066, India; 3 Department of Biomedical Science, University of Sheffield, Sheffield S10 2TN, UK

**Keywords:** Morphogen gradient formation, Wg, Wnt, Protein secretion, Wing epithelium, *Drosophila*, Fz2, Frizzled

## Abstract

Wnts are secreted proteins that regulate cell fate during development of all metazoans. Wnt proteins were proposed to spread over several cells to activate signaling directly at a distance. In the *Drosophila* wing epithelium, an extracellular gradient of the Wnt1 homolog Wingless (Wg) was observed extending over several cells away from producing cells. Surprisingly, however, it was also shown that a membrane-tethered Neurotactin-Wg fusion protein (NRT-Wg) can largely replace endogenous Wg, leading to proper patterning of the wing. Therefore, the functional range of Wg and whether Wg spreading is required for correct tissue patterning remains controversial. Here, by capturing secreted Wg on cells away from the source, we show that Wg acts over a distance of up to 11 cell diameters to induce signaling. Furthermore, cells located outside the reach of extracellular Wg depend on the Frizzled2 receptor to maintain signaling. Frizzled2 expression is increased in the absence of Wg secretion and is required to maintain signaling and cell survival in *NRT-wg* wing discs. Together, these results provide insight into the mechanisms by which robust Wnt signaling is achieved in proliferating tissues.

## Abstract (precis)

**Highlighted Article:** Measuring the direct range of action of the Wg gradient reveals that Wg morphogen signaling in the growing epithelia is regulated by both long-range gradient-dependent and gradient-independent mechanisms.

## INTRODUCTION

Wnts are lipid-modified secreted proteins conserved in all metazoans and play a pivotal role in development and many diseases (Nusse and Clevers, 2017; Zhan et al., 2017). The role of Wnt as a potential long-range signaling molecule has been controversial. Different methods to elucidate the range of extracellular Wnt and to manipulate gradient formation have generated contradictory paradigms for its range of action. On the one hand, direct and indirect visualization approaches can detect Wnt proteins up to several cell distances away from Wnt-producing cells in *C*. *elegans* and *Drosophila* (Neumann and Cohen, 1997; Pani and Goldstein, 2018; Zecca et al., 1996). In addition, studies of Wnt protein carriers, e.g. exosomes and other vesicles, lipoprotein particles and proteinaceous co-factors (Beckett et al., 2013; Greco et al., 2001; Gross et al., 2012; Korkut et al., 2009; Luga et al., 2012; Mulligan et al., 2012; Neumann et al., 2009; Panáková et al., 2005), support a model whereby Wnt proteins can travel over long distances. Furthermore, a broad and graded expression of Wnt target genes with a localized Wnt expression domain has been observed during patterning of the mammalian primary body axis, displaying morphogen-like activities (Aulehla et al., 2003, 2008; Gao et al., 2011; Gavin et al., 1990; Kiecker and Niehrs, 2001; Niehrs, 2010). On the other hand, the long-range morphogen model for Wnt proteins has been challenged by recent studies, showing that juxtacrine Wnt signaling to the neighboring cell via a membrane-tethered Wnt protein is sufficient for proper tissue patterning in *Drosophila* (Alexandre et al., 2014). Furthermore, a primarily short-range distribution of Wnt proteins is observed in the mouse intestinal crypts, mediated by Wnt-membrane association and cell division (Boutros and Niehrs, 2016; Farin et al., 2016), and Wnts are also found on the surface of cytonemes and filopodia (Huang and Kornberg, 2015; Mattes et al., 2018; Stanganello et al., 2015; Stanganello and Scholpp, 2016).

Arguably, the strongest competing models for the mode of action of Wnt proteins have been proposed based on experiments performed in the developing *Drosophila* wing epithelium: the wing imaginal disc. Until ∼84 h after egg laying, Wg is expressed broadly in the developing wing primordium; however, it later becomes restricted to two rows of cells at the dorso-ventral (DV) boundary (Alexandre et al., 2014; Couso et al., 1993; García-García et al., 1999; Ng et al., 1996; Williams et al., 1993). Previous studies have suggested that Wg can spread over several cell distances to directly activate the expression of genes requiring high Wg input, such as *senseless*, and genes requiring low Wg input, such as *Distal-less* (*Dll*), in a concentration-dependent manner (Cadigan et al., 1998; Neumann and Cohen, 1997; Struhl and Basler, 1993; Zecca et al., 1996). However, the exact functional range of Wg remained unknown. Clonal expression of Wg, but not of a membrane-tethered Neurotactin (NRT)-Wg fusion protein, generated a protein gradient and activated target gene expression in distally located cells (Zecca et al., 1996). In contrast to this, a recent study showed that knock-in of NRT-Wg can almost completely replace endogenous Wg, supporting normal tissue patterning, albeit with growth defects (Alexandre et al., 2014), suggesting that Wg spreading is not necessary for normal patterning of wing imaginal discs. These competing models raise two fundamental questions: (1) what is the direct functional range of endogenous Wg protein; and (2) how is the membrane-tethered NRT-Wg able to generate similar wing patterning to endogenous Wg?

Understanding the functional range of Wg protein for patterning the wing epithelium has so far been challenging for two main reasons; first, as mentioned above, *wg* expression changes dynamically from early to late larval stages; and, second, the confounding effect of parallel tissue growth, whereby proliferation of cells leads to their movement away from the localized source of the extracellular protein gradient (Dekanty and Milán, 2011). Although target gene expression in these cells would be expected to reduce gradually, it was, however, shown that expression of Wg target genes that require a low-level of signaling were in fact sustained in the absence of Wg (Piddini and Vincent, 2009). This suggests that, although largely unknown, mechanisms may exist to compensate for the decreasing concentration of Wg experienced by cells moving out of the Wg gradient.

In this study, we captured Wg proteins at a distance from the producing cells and measured the direct range of action of the Wg gradient by generation of single cell clones expressing the Frizzled2 (Fz2) receptor. By this means, we demonstrate that Wg can spread over at least 11 cells to activate signaling. Surprisingly, we also find that, at the extracellular level, membrane-tethered NRT-Wg is not restricted to the producing and adjacent receiving cells but can also be detected further away. We propose that persistence of NRT-Wg protein in the receiving cells, perhaps owing to the properties of the NRT protein, could contribute to its ability to pattern the wing imaginal disc. Furthermore, we show that cells that have moved out of the range of a functional Wg gradient then require the Fz2 receptor to maintain the expression of low-threshold Wg target genes, which is important for the survival of cells in *NRT-wg* discs. Altogether, our study demonstrates that Wg morphogen signaling in the growing epithelia is regulated by both long-range gradient-dependent and gradient-independent mechanisms.

## RESULTS

### Wg acts up to a distance of 11 cells

**Fig. 1.**
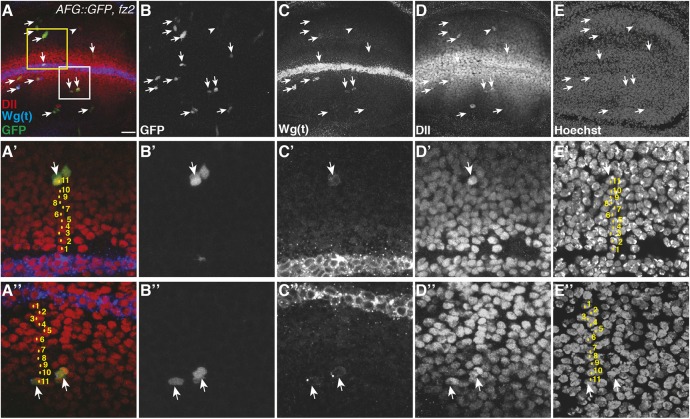
**Wg can be detected up to 11 cell diameters from the Wg-producing cells.** (A-E) Single cell flip-out clones overexpressing Fz2 were generated in wing imaginal discs (marked with GFP, arrows). 24 h after the heat-shock to induce Fz2-expressing single cell clones, total Wg staining [marked Wg(t)] (see Materials and Methods) shows clones with accumulation of Wg (C, images show projection of several confocal slices). Dll levels were modestly upregulated in these GFP-positive cells, while very few cells also show Dll upregulation without the apparent expression of GFP (B, arrowhead). (A′-E″) Enlarged images of the areas marked in A with a yellow box (shown in A′-E′) and a white box (shown in A″-E″). A merge of three confocal slices was used to count the number of nuclei between the Fz2-overexpressing clone and Wg-producing cells. GFP-positive cells (A′,A″,B′,B″, arrows) showing higher levels of Wg (C′,C″) and Dll (D′,D″) are at a distance of ∼11 nuclei from the Wg-producing cells (nuclei marked with yellow dots). Scale bar: 20 μm. *n*≥4 discs (see also Fig. S1).

We first set out to investigate the direct range of a functional Wg gradient. Previous studies have shown that overexpression of Fz2 causes Wg stabilization at the cell surface (Bhanot et al., 1996; Cadigan et al., 1998; Piddini et al., 2005). We made use of this observation to probe how far from the DV boundary the Fz2 receptor could capture Wg protein and enhance Wg-dependent signaling output. We generated single cell Fz2-overexpression clones using the heat-shock inducible flip-out system. In order to control for the effect of cell proliferation on the accuracy of determining the Wg range, we induced the expression of Fz2 only at mid-third-instar larval stage and reduced the growth rate by keeping larvae at 18°C after the heat shock. By these means, we recovered single cell clones of Fz2 (marked by GFP) in the late third-instar wing disc (Fig. 1A-E, arrows; and Fig. S1). These GFP-marked cells showed accumulation of Wg (Fig. 1C-C″) and a higher level of Dll expression (Fig. 1D-D″) than surrounding control cells. We determined that these Wg-accumulating cells on either side of the DV boundary can be found up to 11 cell distances away from the DV boundary (Fig. 1A′,A″,E′,E″, yellow dots). GFP-positive cells present much further from the DV boundary did not show either accumulation of Wg or Dll upregulation (Fig. S1E″-H″). We further confirmed these results by analyzing Wg accumulation in single cell clones overexpressing a GPI-linked N-terminal fragment of Fz2, which acts as a dominant negative. Consistent with the Fz2 overexpression, these single cell clones accumulated Wg protein; however, they did not upregulate Dll expression (Fig. S2A-D), suggesting that Dll activation required the formation of functional Wg-Fz2 complexes.

We next asked whether the accumulation observed in the Fz2-overexpressing cells could be due to previous expression of Wg in these cells. To test this, we analyzed whether HA-tagged NRT-Wg, which is not secreted by the cells (Alexandre et al., 2014), will accumulate in Fz2-expressing cells at a similar distance observed with normal Wg. As shown in Fig. S3A-E and F-J, Fz2 expressing cells at four cell distances from the DV boundary show accumulation of HA-positive and thus NRT-Wg-positive puncta; however, clones at either seven or nine cell distances did not show accumulation of NRT-Wg. These results rule out that normal Wg accumulation observed at 11 cell distances was due to its previous expression and protein perdurance. Taken together with previous observations that Wg expression from mid-third-instar stages is restricted to the DV boundary (Alexandre et al., 2014; Couso et al., 1993; García-García et al., 1999; Ng et al., 1996; Williams et al., 1993), these results suggest that endogenously secreted Wg can reach a distance of up to 11 cells to directly regulate transcriptional targets.

### Membrane-tethered Wg protein is detected in Wg-receiving cells

**Fig. 2.**
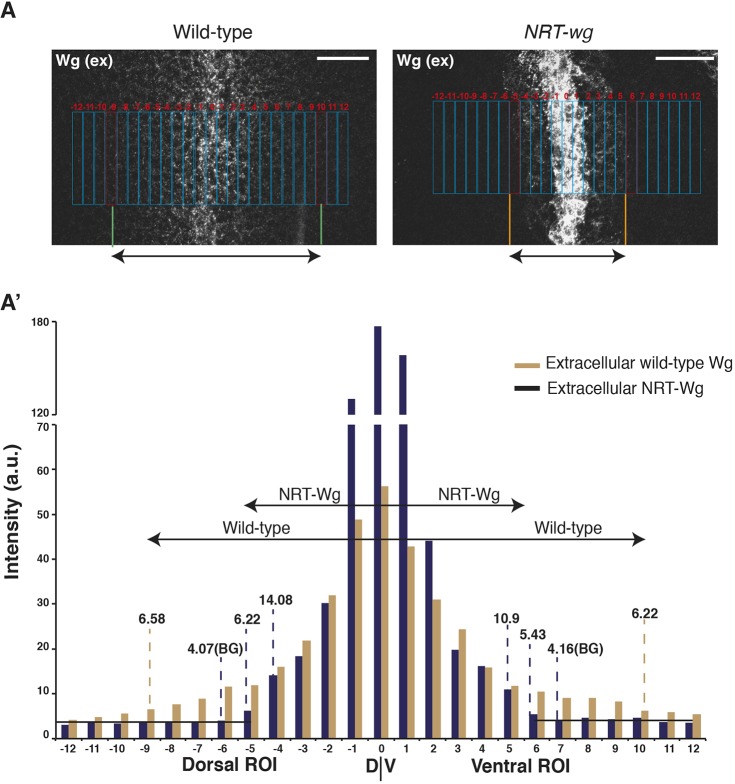
**Comparison of extracellular Wg staining of wild-type and *NRT-wg* wing imaginal discs.** Quantification of the extracellular Wg in homozygous wild-type or *NRT-wg* discs (images also shown in Fig. 3A′,B′). (A) Distribution of extracellular Wg in the homozygous *NRT-wg* or wild-type (*w^1118^*) discs was divided into 25 rectangular ROIs across the DV boundary (−12 to DV to 12), where each ROI represents approximately a single row of cells. (A′) Intensity was measured for each ROI separately and for ROIs on either side of the DV boundary, where the intensity was higher than the background signal (BG, ∼4) were selected for determining the range of extracellular Wg on the *NRT-wg* discs (from segment −5 to between 5 and 6). ROIs showing similar intensities were used for determining the range of extracellular Wg on wild-type discs (from region −9 to 10). Images were processed and analyzed using ImageJ under identical conditions. Scale bars: 20 μm.

The results presented above raise the question of how can NRT-Wg, which is thought to be restricted to the expressing cells, induce a similar patterning of the tissue as wild-type Wg? Alexandre et al. (2014) showed that low levels of NRT-Wg protein, analyzed using total Wg staining, could be detected in cells presumed to be non-Wg expressing cells, although secretion of NRT-Wg was ruled out. We hypothesized that additional quantitative analysis of the reach of extracellular NRT-Wg when compared with the extracellular wild-type Wg and of the extent of target gene expression will provide a better understanding of their signaling activity range. To address this, we performed extracellular Wg staining on either wild-type or homozygous *NRT-wg* third-instar discs and imaged them using identical settings. We then analyzed the distribution of extracellular Wg by dividing the images into small regions of interests (ROIs) (corresponding approximately to one row of cells) across the DV boundary and measured fluorescent intensity in each ROI (Fig. 2A,A′). At this stage of disc development, it is expected that NRT-Wg, being membrane tethered, will be restricted to the two stripes of *wg*-expressing cells at the DV boundary. However, the extracellular NRT-Wg protein was detected over a broader domain covering several receiving cells (Fig. 2A′). Furthermore, consistent with the higher levels of total NRT-Wg protein detected previously (Alexandre et al., 2014), the levels of extracellular NRT-Wg protein were also much higher in the producing cells when compared with endogenous Wg.

**Fig. 3.**
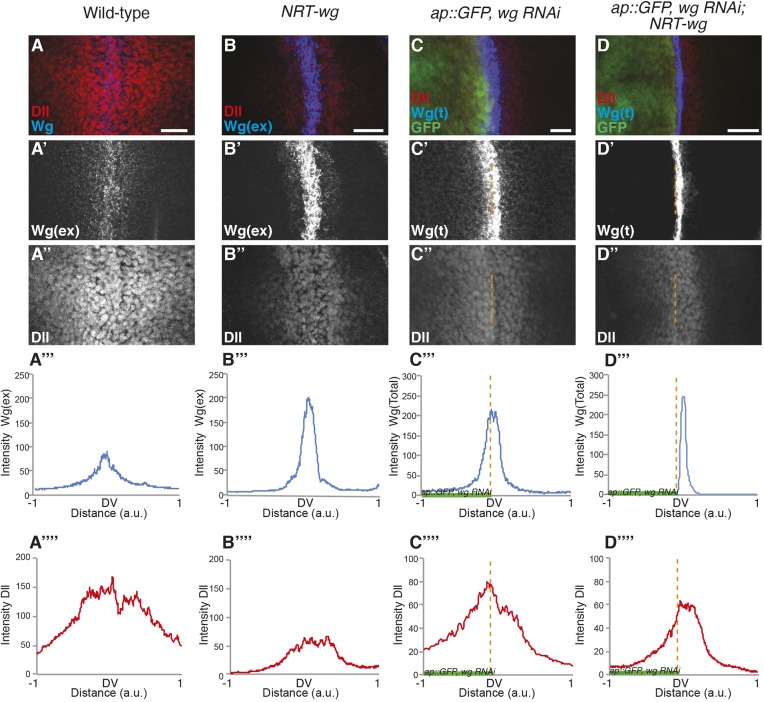
**Presence of membrane-tethered Wg in the receiving cells rescues signaling.** (A-B″) Representative images of extracellular Wg and Dll staining on either wild-type (A-A″) or *NRT-wg* homozygous (B-B″) discs imaged with the same confocal settings (*n*=4). (A‴,A″″,B‴,B″″) Graphs show intensity profiles with an *x*-axis similar in scale to the images. Images in A′ and B′ are reproduced from Fig. 2A. (C-D″) Total Wg and Dll staining on discs with depletion of Wg in dorsal compartment (left, marked by GFP and dotted orange lines) with *ap-Gal4, UAS-GFP/UAS-wg-RNAi* in either wild-type (C-C″) or *NRT-wg* homozygous (D-D″) background; *n*=4. (C‴,C″″,D‴,D″″) Graphs show intensity profiles with an *x*-axis similar in scale to the images. a.u., arbitrary unit. Scale bars: 20 μm.

Next, we analyzed the range of Dll expression in these discs. Compared with the wild-type discs, Dll expression was narrower in the *NRT-wg* discs (Fig. 3A,B), consistent with previous observations (Alexandre et al., 2014). We also observed that Dll was expressed at higher levels in cells positive for NRT-Wg, compared with cells where extracellular NRT-Wg was below detectable levels (Fig. 3A″-B″ and Fig. S4A″-B″). This indicates that the expression of target genes in *NRT-wg* discs could be directly regulated by the presence of NRT-Wg protein on the surface of cells outside the normal Wg expression domain.

### Retention of membrane-tethered Wg in the receiving cells rescues signaling defects

We next asked whether the broader *wg* promoter activity detected during early larval stages contributed towards the presence and effect of both endogenous wild-type Wg and NRT-Wg in the receiving cells at later stages (no longer expressing *wg* mRNA). To this end, we continually depleted Wg or NRT-Wg specifically in the dorsal compartment of the disc by expressing *wg* RNAi under the control of the *ap-Gal4* driver (Fig. 3C,D and Fig. S4C,D, left side of the disc also marked by GFP), leaving the ventral compartment and the ventral stripe of Wg-producing cells unaffected. In wild-type wing discs, where Wg is secreted from the ventral stripe of producing cells and spreads over several cells in both directions, we were able to detect Wg-positive puncta in *wg* RNAi-expressing cells of the dorsal compartment using either total Wg (Fig. 3C,C′,C″′ and Fig. S4C,C′, left side of the disc also marked by GFP) or extracellular Wg staining methods (Fig. S4E). Accordingly, Dll expression in the dorsal compartment was similar to the ventral control compartment (Fig. 3C″,C″″ and Fig. S4C″). In contrast, expression of *wg* RNAi in the dorsal compartment of *NRT-wg* homozygous discs led to a strong reduction of both total and extracellular levels of NRT-Wg protein (Fig. 3D,D′,D″′ and Fig. S4D,D′) (Fig. S4F). Consequently, this led to a strong reduction in Dll expression in the dorsal compartment (Fig. 3D″,D″″ and Fig. S4D″). These experiments suggest that, unlike wild-type Wg, the presence of NRT-Wg in the receiving cells requires expression of *NRT-wg* during earlier stages and likely reflects longer persistence of NRT-Wg protein in these cells.

### Fz2 receptor activity maintains expression of long-range Wg target genes

Because, in late third-instar discs, Dll is expressed beyond the 11 cell diameter range of the Wg protein gradient, we next asked how Dll expression can be maintained in the absence of extracellular Wg. One way of maintaining Wnt signaling could be a modulation of the expression of feedback regulators, which can provide either positive or negative input at various levels of the pathway. To this end, we turned our attention to the Fz2 receptor, which is a positive regulator of the pathway that is transcriptionally repressed by Wg signaling (Cadigan et al., 1998). Reducing Wg function in discs during late larval stages causes upregulation of Fz2 mRNA (Cadigan et al., 1998), whereas Dll expression remained unaffected (Piddini and Vincent, 2009). Therefore, we hypothesized that Dll levels could be maintained by the increased amount of Fz2 receptors in distal cells.

**Fig. 4.**
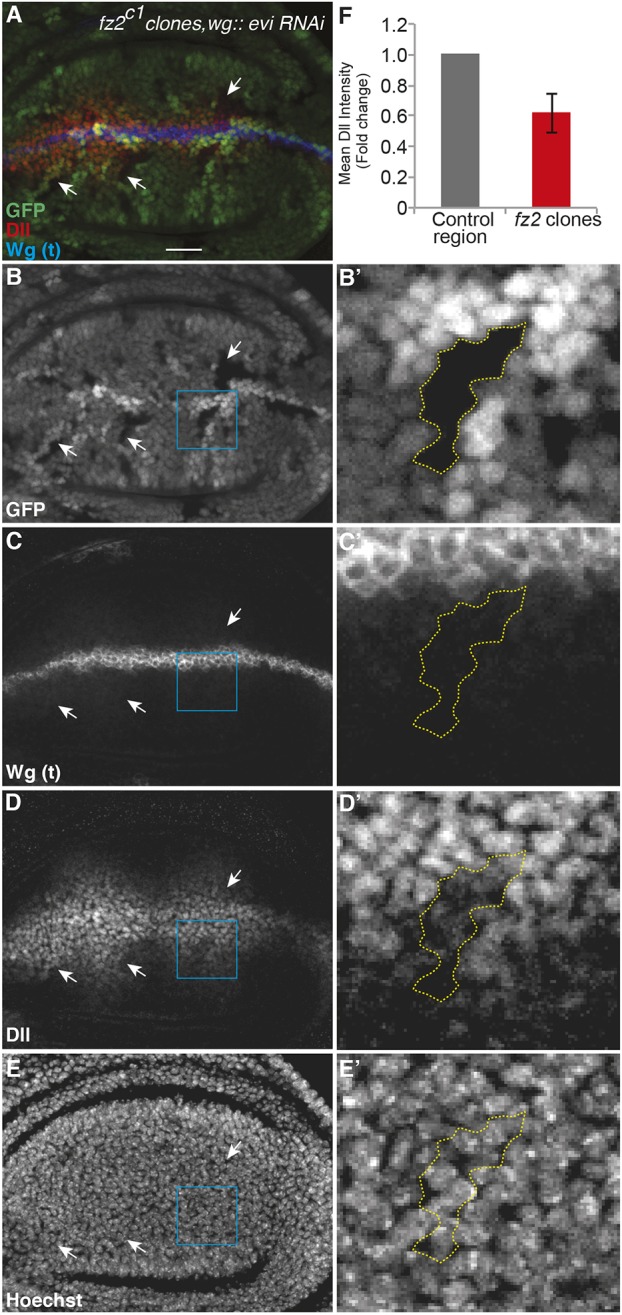
**Fz2 is required for the maintenance of Dll expression in the absence of Wg secretion.** Genotype of the disc shown in this figure is *Ubx-FLP/+; wg-Gal4/UAS-evi-RNAi; fz2^c1^, ri, FRT2A/ubi-GFP FRT2A.* (A-E) *fz2^c1^* clones (marked by absence of GFP) were generated in discs also expressing *evi-RNAi*, leading to accumulation of Wg in the producing cells due to a block in Wg secretion (C). Dll expression is reduced inside the *fz2^c1^* clones (D, arrows). (B′-E′) Enlargements of the regions outlined in B-E. **(**C′,D′**)** Dll expression is reduced inside the clone close to the receiving cells. (E′) Hoechst staining shows presence of nuclei inside the clone as in neighboring control cells. (F) Quantification of Dll intensity with in the *fz2^c1^* clones and the neighboring control region at the same distance from the DV boundary (*n*=12 clones). Scale bar: 20 μm.

First, we confirmed the upregulation of Fz2 at the protein level using antibody staining. Blocking Wg secretion by depletion of the Wnt cargo-receptor Evenness interrupted (Evi; also known as Wntless) (Bartscherer et al., 2006; Bänziger et al., 2006; Goodman et al., 2006) in the posterior compartment led to a modest increase in Fz2 protein levels (Fig. S5A,A′, compare arrowhead with arrow). Interestingly, in these cells Dll remained expressed (Fig. S5A″). Next, we asked whether the increase in Fz2 protein levels upon blocking Wg secretion supports maintenance of Dll expression. To address this, we used the *fz2^c1^* null mutants (Chen and Struhl, 1999) to generate *fz2* loss-of-function clones in discs expressing *evi* RNAi under the control of either *wg-Gal4* or *en-Gal4*, leading to a loss of secreted Wg either in the entire disc or the posterior compartment, respectively*.* As shown in Fig. 4A-F (for *wg-Gal4>evi RNAi*) and Fig. S5B,C (for *en-Gal4>evi RNAi*), we observed a significant loss of Dll expression (Fig. 4D, arrows; Fig. S5B″,C″, arrows) in *fz2* mutant clones (marked by loss of GFP) compared with the neighboring GFP-positive control tissue. Therefore, these data demonstrate that, in the absence of the extracellular Wg gradient upon Evi depletion, cells upregulate Fz2 levels, which is required for the maintenance of Dll expression.

Fz2 acts redundantly with Frizzled1 (Fz1) in canonical Wnt signaling (Bhanot et al., 1999; Bhat, 1998; Chen and Struhl, 1999; Kennerdell and Carthew, 1998; Müller et al., 1999). We next asked whether Fz2, which is expressed at higher levels in cells away from the DV boundary, has a non-redundant role with Fz1 in maintaining Dll expression. To test this, we analyzed Dll expression in *fz2* mutant clones either close to or distant from the DV boundary in otherwise wild-type discs (Fig. S5D-F). Under this condition, *fz2* clones, which were close to the DV boundary (marked by white box in Fig. S5D), showed no effect on Dll levels (Fig. S5E-E‴), as previously shown (Chen and Struhl, 1999). However, in contrast, *fz2* clones further away from the DV boundary (marked by yellow box in Fig. S5D) showed reduced levels of Dll expression compared with control cells at the same distance (Fig. S5F-F‴, compare arrows marking clones with arrowhead marking a control cell). However, *fz1* clones showed no difference in Dll levels at both short and long range (Fig. S6A-D). Notably, in wing discs where Wg secretion was blocked, remaining Dll expression was reduced in *fz2* mutant clones irrespective of the location of the clone from the DV boundary (Fig. 4B′-E′), supporting the conclusion that Wg-independent expression of Dll is Fz2 dependent.

### Higher levels of Fz2 protein in *NRT-wg* discs contribute to its rescue effects

**Fig. 5.**
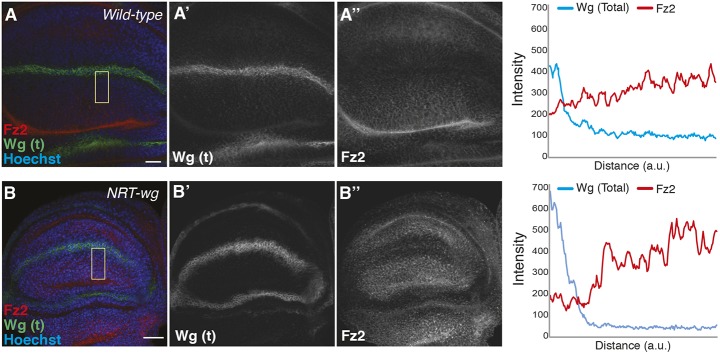
**Fz2 levels are increased in cells beyond the reach of NRT-Wg.** (A-B″) Representative images of Fz2 and Wg staining on either wild-type (A-A″) or *NRT-wg* homozygous (B-B″) discs imaged with the same confocal settings. Graphs on the right show intensity profiles across the yellow boxes in A and B. a.u., arbitrary unit. Scale bars: 20 μm.

Based on these results, we hypothesized that Fz2 may also play a role in maintenance of long-range signaling in *NRT-wg* discs, and thus may be required for the survival of *NRT-wg* flies. We first analyzed the expression of the Fz2 receptor in the *NRT-wg* discs, where we find higher levels in *NRT-wg* homozygous discs compared with the control (Fig. 5A,B). Next, we tested whether Fz2-mediated maintenance is essential for the survival of *NRT-wg* flies. To this end, we analyzed Fz2 loss of function (LOF) by combining *fz2^c1^* null mutants with the *fz2* deficiency line (*fz2* LOF) in the *NRT-wg* homozygous flies. Previously, it has been shown that *NRT-wg* homozygous flies eclosed at a near-normal rate when grown separately (Alexandre et al., 2014); thus, we also separated control, *fz2* knockout, *NRT-wg* and *fz2* LOF; *NRT-wg* larvae to analyze eclosion. We observed a significant reduction in the eclosion of *fz2* LOF; *NRT-wg* compared with either *NRT-wg* or *fz2* LOF (Fig. S7). These results suggest that Fz2-mediated maintenance is important for the overall survival of *NRT-wg* flies.

**Fig. 6.**
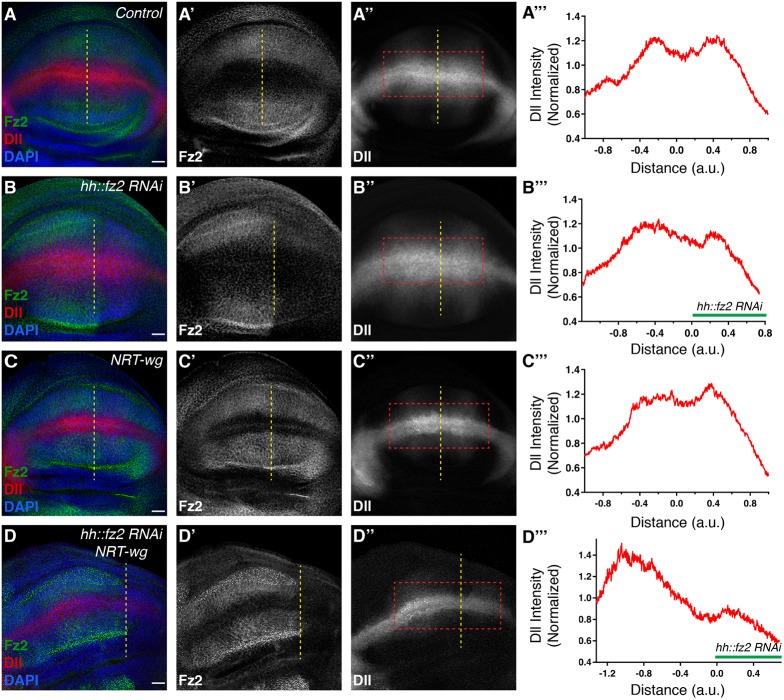
**Fz2 is required for the maintenance of Dll expression in *NRT-wg* background.** (A-A″) Fz2 and Dll expression (measured using an endogenous Dll reporter) in control wing imaginal disc (*n*=7). Yellow dotted line separates anterior (left) and posterior (right) compartments, identified by the anterior zone of non-proliferating cells (ZNC). (B-B″) Fz2 and Dll expression upon *fz2* knockdown in the posterior compartment with *hh-Gal4* (*n*=10); Dll levels remain unaffected. Anterior and posterior compartments are marked by Fz2 levels. (C-C″) Fz2 and Dll expression in *NRT-wg* (*n*=8). Anterior-posterior compartments are marked by the anterior ZNC. (D-D″) Fz2 and Dll expression in *NRT-wg* background upon *fz2* knockdown in the posterior compartment (*n*=8), Dll levels are reduced in the posterior region. Anterior and posterior compartments are marked by Fz2 levels. (A‴-D‴) Graphs depict the Dll intensity profile across the red box for the respective genotypes. a.u., arbitrary unit. Scale bars: 20 μm.

**Fig. 7.**
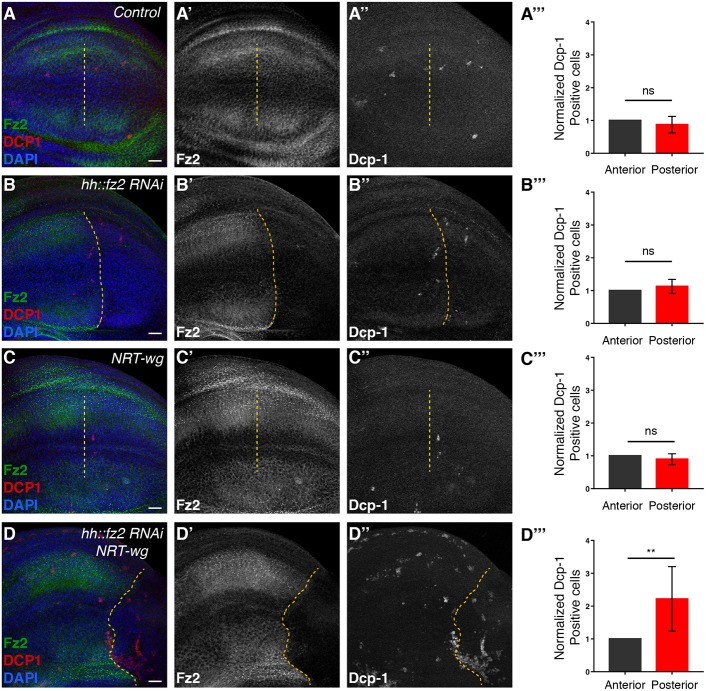
**Depletion of Fz2 leads to higher cell death in *NRT-wg* discs.** (A-A″) Fz2 and Dcp-1 (cell death marker) expression in control wing imaginal disc (*n*=8). Yellow dotted line separates anterior (left) and posterior (right) compartments, identified by the anterior zone of non-proliferating cells (ZNC). (B-B″) Fz2 and Dcp-1 expression upon *fz2* knockdown in the posterior compartment with *hh-Gal4*; Dcp-1 levels remain unaffected (*n*=11). Anterior and posterior compartments are marked by Fz2 levels. (C-C″) Fz2 and Dcp-1 expression in *NRT-wg* (*n*=8). Anterior-posterior compartments are marked by the anterior ZNC. (D-D″) Fz2 and Dcp-1 expression upon *fz2* knockdown in the posterior compartment of *NRT-wg* discs; Dcp-1 levels are increased in the posterior region (*n*=8, *P*=0.0036). Anterior and posterior compartments are marked by Fz2 levels. (A″′-D″′) The graphs represent normalized Dcp-1-positive cells in the posterior compartment compared with the anterior compartment for the respective genotypes. Unpaired Student's *t*-test was applied for statistical analysis. ns, not significant; **P<0.01. Scale bars: 20 μm.

To further analyze the importance of Fz2-mediated maintenance, we depleted Fz2 in the posterior compartment of the control and *NRT-wg* discs. Expression of *fz2* RNAi using *hh-Gal4* in an otherwise wild-type background showed no change in the Dll expression when compared with the control (Fig. 6A,B) and no effect on cell survival, as observed by Death caspase-1 (Dcp-1) staining (Fig. 7A,B). However, depletion of Fz2 in the *NRT-wg* background led to reduction in the levels of Dll (Fig. 6C,D) and a strong increase in cell death, in regions mostly away from the DV boundary (Fig. 7C,D, also see Fig. S7B, arrows). In some samples, the posterior compartment expressing *fz2* RNAi also appeared to be reduced in size in the *NRT-wg* background. In contrast to this, depletion of Fz1 in either a control background or *NRT-wg* background did not affect Dll levels (Fig. S8A,B) and also did not show an increase in cell death (Fig. S8C,D). Collectively, these data demonstrate that although short-range signaling is Wg dependent and can be mediated by either Fz2 or Fz1, the long-range expression of Dll is Wg independent and regulated by Fz2. Moreover, this indicates that Fz2-mediated maintenance signaling, which is non-redundant with Fz1, plays an important role in cell viability and in the overall survival of *NRT-wg* animals.

## DISCUSSION

How secreted proteins influence patterning decisions at a distance has been a long-standing and fundamental question in developmental biology (Gierer and Meinhardt, 1972). It has been proposed that signaling molecules can act as morphogens by being secreted from a localized source and form gradients across a tissue to induce patterning decisions in a concentration-dependent manner (Gierer and Meinhardt, 1972; Rogers and Schier, 2011; Wolpert, 1969, 1971). However, the biochemical nature of these gradients and their functional ranges are in many cases not well understood. It also remains largely unresolved how cells moving away from the source of such ‘morphogen’ signaling factors maintain their identity in a rapidly growing tissue.

In this study, we provide experimental evidence that Wg/Wnt signaling in target cells of the developing wing epithelium is regulated by the combined inputs from: (1) a long-range Wg activity detected at an 11-cell distance; and (2) the maintenance of Wg/Wnt signaling by the Fz2 receptor in cells exposed to gradually decreasing levels of Wg.

### Determining the signaling range of Wg in the developing *Drosophila* wing

Although almost four decades have passed since the discovery of Wnt, the mechanism by which Wnt proteins mediate long-range effects still remains poorly understood. Contradictory models proposing either a direct long-range spreading or juxtacrine signaling of Wnt proteins to the immediate neighboring cell have been discussed (Pani and Goldstein, 2018; Neumann and Cohen, 1997; Zecca et al., 1996; Alexandre et al., 2014; Bartscherer et al., 2006; Morata and Struhl, 2014). In this study, by overexpression of the Fz2 receptor in single cell clones, we demonstrate that Wg, but not NRT-Wg, can be captured at a distance of at least 11 cells from its origin. Single cell clones were generated at a late stage of third-instar development when Wg expression is restricted to a narrow stripe at the DV boundary. These experiments also showed that capturing of Wg by Fz2 leads to a cell-autonomous increase in Dll expression, indicating that captured extracellular Wg is functional. Taken together, our results suggest that secreted Wg is active up to a distance of 11 cells in developing wing imaginal discs, concordant with its visualization in the extracellular space.

### Persistence of membrane-tethered Wg compensates for its inability to act at a distance

**Fig. 8.**
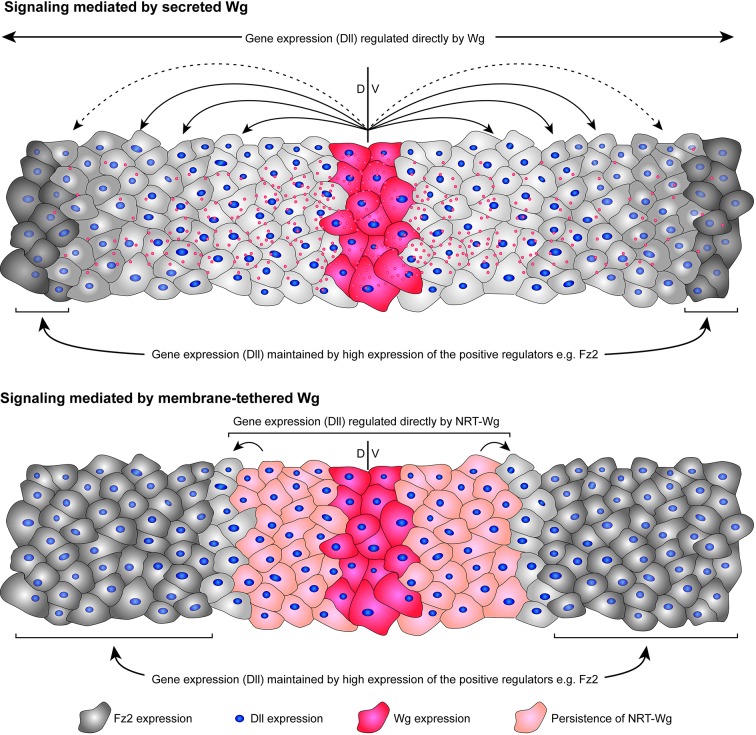
**Model representing the mechanism of signaling via secreted Wg that can act up to a distance of at least 11 cells.** Cells beyond the range of secreted Wg can maintain Dll expression by upregulating positive regulators, e.g. the Fz2 receptor. Persistence of membrane-tethered NRT-Wg mediates signaling up to a few cell distances, beyond which cells depend on higher levels of Fz2 to maintain target gene expression.

If Wg proteins are able to travel long distances and mediate signaling, then how can a wild-type, secreted Wg protein be functionally replaced by a membrane-tethered version? Our experiments provide several lines of evidence that point towards a longer persistence of NRT-Wg protein when compared with wild-type Wg, as also previously discussed by Morata and Struhl (2014). The persistence of NRT-Wg, either due to higher stability of the NRT-Wg protein, probably owing to its inability to be secreted by the cells or owing to low-level expression, leads to the presentation of the ligand in cells that, under wild-type conditions, would not express but receive a Wg signal (Fig. 8). First, immunofluorescence staining of extracellular NRT-Wg protein showed that it is not limited to Wg-producing cells but can also be detected in receiving cells, which, presumably as a consequence, express high-levels of the Wg target gene *Dll*. Second, when we depleted NRT-Wg by RNAi in the dorsal compartment of *NRT-wg* homozygous discs, this led to a complete removal of NRT-Wg dorsally, whereas its expression appeared normal in the ventral compartment. In contrast, in wild-type Wg discs, we observed a gradient of Wg and a similarly reduced expression pattern in both the RNAi knockdown and control compartments. Although it is possible that NRT-Wg protein is transferred from one cell to other via cell division, as previously suggested for Wg in *Drosophila* embryo (Pfeiffer et al., 2000) and for mammalian Wnt3 (Farin et al., 2016), our results suggest that the wild-type Wg protein gradient observed in wing discs is generated by secretion and spreading, and not due to cell division and cell lineage-based expression. Furthermore, in contrast to the depletion of NRT-Wg, Dll expression remained unchanged upon depletion of normal Wg. These results suggest that, under normal conditions, the early broad expression of Wg observed in the developing wing until 84 h after egg laying (Alexandre et al., 2014; Couso et al., 1993; García-García et al., 1999; Ng et al., 1996; Williams et al., 1993) does not contribute to the target gene expression at later stages, which is regulated by the later Wg expression that is restricted to the DV boundary region. Interestingly, recent studies showed that NRT-Wg is unable to replace endogenous Wg for proper patterning of the Malpighian tubules (Beaven and Denholm, 2018), and the adult intestine and visceral muscles (Stewart et al., 2019; Tian et al., 2019). These studies support the requirement of long-range distribution of Wg for proper patterning of tissues, and also support a model whereby a rescue effect of NRT-Wg observed in tissues such as the wing imaginal disc may depend on the tissue-specific compensatory mechanisms, e.g. maintenance of signaling.

### Maintenance of long-range target genes by Fz2

Although our results show that target gene expression at a distance of 11 cells can be under the direct control of a Wg gradient, how signaling activity of Wg beyond this range is established remained largely unresolved. Previous studies have suggested that the expression of Wg target gene *vestigial*, once initiated by boundary enhancer, could be maintained with the help of Polycomb-group (PcG) response elements present nearby (Pérez et al., 2011). Similarly the expression of Dll can be maintained after initiation by auto-regulatory mechanisms via cis-regulatory elements in a Wg-independent manner (Estella et al., 2008; Galindo et al., 2011; Piddini and Vincent, 2009). However, the enhancers identified were found to be active mainly in the leg imaginal discs and did not explain the expression of Dll observed at long range in the wing discs. In this study, we show that the Fz2 receptor, which is transcriptionally repressed by Wg signaling, is upregulated upon either block of Wg secretion or in *NRT-wg* discs in cells where Dll expression is maintained. Removal of Fz2 from these cells led to further reduction of Dll levels, indicating that Fz2 is required for the maintenance of Dll expression in the absence of continuous input from extracellular Wg. More importantly, removal of Fz2 in *NRT-wg* flies showed higher cell death and overall lethality, suggesting that Fz2-mediated maintenance is functionally important and contributed towards the survival of *NRT-wg* flies.

Furthermore, *fz2* mutant clones generated in otherwise wild-type discs showed reduced levels of Dll in clones found only at long range and not at short range. Fz1, unlike Fz2, is uniformly expressed in the discs, therefore loss of Fz2 at short range would be rescued by Wg-dependent signaling via Fz1. However, Fz1 is unable to rescue long-range Dll expression in Fz2 clones, indicating that this expression of Dll is not regulated directly by extracellular Wg. This suggests that cells at long range have moved out of the functional Wg gradient, which led to an increase in Fz2 expression that further maintains Dll expression (Fig. 8). Therefore, our data reveal a new non-redundant role for the Fz2 receptor in maintaining Wnt signaling. Interestingly, previous studies have proposed mechanisms by which Frizzled receptors can activate signaling in a ligand-independent manner. For example, Fz receptor dimerization was shown to induce ligand-independent signaling in *Xenopus* (Carron et al., 2003). Furthermore, a recent study has shown a membrane protein known as TMEM59 can promote ligand-independent signalosome formation and activation of Wnt signaling (Gerlach et al., 2018). Whether Fz2-mediated maintenance follows similar mechanisms remains to be studied.

Feedback regulation in gradient signaling is a common mechanism used in a variety of growing tissues by various other signaling molecules, e.g. in the case of Dpp (Ben-Zvi et al., 2011; Vuilleumier et al., 2010). Positive regulators are transcriptionally repressed whereas the negative-feedback genes are transcriptionally activated. This allows cells moving out of the functional range of the Wg gradient to gradually reduce the levels of negative regulators, e.g. a decoy receptor Frizzled3 (Sato et al., 1999) and increase the amount of positive regulators, e.g. Fz2 (Cadigan et al., 1998) and the Wg co-receptor Arrow (Wehrli et al., 2000). Like a balancing pole used for tight-rope walking, this underlying mechanism in a rapidly growing tissue enables cells to balance signaling at different positions along the Wg concentration gradient.

### Conclusions

In summary, our data support a model whereby the endogenous Wg protein can travel over distances of several cells to induce a Wnt signaling cascade. Although the underlying biochemical mechanisms – such as secretory vesicles, proteinaceous particles or filopodia – remain unknown, our data indicate that developing tissues retain a high degree of plasticity to respond to changes in extracellular cues with multiple interacting mechanisms. Maintenance and other compensatory mechanisms may play an important role in buffering fluctuations resulting in the proper patterning of the wing epithelium. It will be interesting to understand the mechanism of receptor-mediated maintenance of Wg signaling in more detail and to identify additional maintenance mechanisms. This study also indicates that genome-engineered variants of proteins might lead to unexpected compensatory mechanisms *in vivo*.

## MATERIALS AND METHODS

### *Drosophila* genetics

The following *Drosophila* stocks were used: *wg-GAL4* (2nd chr*.,* a gift from S. Cohen, University of Copenhagen, Denmark), *hh-Gal4 3rd chr.* (Tanimoto et al., 2000), *ap-GAL4* 2nd chr. (Montagne et al., 1999) and *en-GAL4, UAS-GFP* 2nd chr. (Thompson and Cohen, 2006), *hs-Flp* on 1st chr. and U*AS-GFP* in 3rd chr. (gifts from A. Teleman, German Cancer Research Center, Heidelberg)*. fz2^C1^, ri, FRT2A* (Chen and Struhl, 1999), *fz^p21^* (a functional null allele) (Jones et al., 1996), *ubi-GFP FRT2A* (a gift from A. Gould, Francis Crick Institute, London, UK), *RIV-FRT-NRT-HA-wg-FRT-wg* and *NRT-HA-Wg* (Baena-Lopez et al., 2013), *evi^2^* (Bartscherer et al., 2006)*. UAS-Fz2* (BDSC, 41794) *UAS-Fz2(ECD)-GPI* (dominant-negative Fz2) (BDSC, 44221), ubi-GFP FRT80 (BDSC, 1620), *Df(3L)fz2* (BDSC, 6754) and *Actin5C-FRT-CD2-FRT-Gal4* (BDSC, 4779) lines were obtained from Bloomington Stock Center (Bloomington, IN, USA). *UAS-wg-RNAi* (GD-ID 13352), *UAS-fz2-RNAi* (KK-ID 108998), *UAS-fz1-RNAi* (KK-ID 105493) and *UAS-evi-RNAi* (KK-ID 103812) lines were obtained from Vienna *Drosophila* RNAi Center. All crosses were reared on standard culture medium at 25°C, except where specifically mentioned.

### Generation of *dTomato-Dll* flies

To generate a transcriptional reporter for *Dll* expression, the coding sequence for the red fluorophore dTomato was inserted 5′ of the *Dll* coding region by CRISPR-assisted homology-directed repair. To avoid a functional impairment of Dll by the fluorescent tag, the coding sequences for dTomato and Dll were separated by a T2A cleavage signal. CRISPR-mediated knock-in of dTomato was performed as previously described (Port et al., 2014). Briefly, two sgRNAs targeting two sites in close proximity of the 5′ end of the *Dll* open reading frame (CCTCCCAGCACCCCCCAGTC and CTTGGGCGTGTGCGGGGCGT) were cloned into pCFD5 following the protocol described previously (Port and Bullock, 2016). The donor plasmid comprised homology arms flanking the cut site of ∼1 kb and a sequence encoding tandem-dimeric (td) Tomato and T2A in-frame with the *Dll* ORF. The homology arms were amplified by PCR from genomic DNA from *nos-Cas9* flies (Port et al., 2014) using primers dll5HRarmfwd (GACGGTATCGATAAGCTTGATATCGAATTCccgagaaaaccggttgcaaaccac), dll5HRarmrev (GACCTCCTCGCCCTTGCTCACCATGGATCCCATGTTCGACTGGGGGGTGCTGGGAG), dll3HRarmfwd (TGCGGTGACGTCGAGGAGAATCCTGGTCCAATGGACGCCCCTGACGCCCCGCACACG) and dll3HRarmrev GAGCTCCACCGCGGTGGCGGCCGCTCTAGAttcagtcttagtcgatttggctgag). The sequence encoding tdTomato:T2A was ordered as a gBlock from Integrated DNA Technologies and amplified by PCR with primers tdTom5fwd (GGATCCATGGTGAGCAAGGGCGA) and tdTom5rev2 (ACGTCACCGCATGTTAGCAGACTTCC). The three PCR products were assembled with pBlueskript-SK+ linearized with EcoRI and XbaI by Gibson Assembly (New England Biolabs) following the protocol provided by the manufacturer. Donor plasmid (400 ng/µl) and sgRNA plasmid (100 ng/µl) were injected into *nos-cas9* transgenic embryos. Offspring were screened for the presence of red fluorescence and ends-out insertion of the donor cassette was confirmed by PCR-based genotyping using primers outside the homology arms (Dll_KI_geno1, AGTATTGGTGCGGTTTCTGCACCAC; Dll_KI_geno2 CATAAAGATGGAAAGCTGACGGCAAC). The correct sequence of the PCR amplicons was confirmed by Sanger sequencing. Surprisingly, genotyping revealed the presence of a single dTomato-coding sequence at the *Dll* locus, presumably reflecting recombination of the two repetitive sequences in tdTomato. dTomato fluorescence is detected in the known Dll expression patterns in wing and leg imaginal discs and responds to perturbations in Wnt signaling in wing imaginal discs. Dll is located at the distal tip of chromosome 2 and not efficiently balanced by common 2nd chromosomal balancers.

### Antibodies

The following antibodies were used: mouse anti-Wg (4D4s, obtained from Developmental System Hybridoma Bank) used 1:5 for extracellular and 1:50 for total staining; rabbit anti-Dll 1:200 (a gift from S. Carroll, University of Wisconsin-Madison, USA), rabbit anti-Dcp-1 1:300 (obtained from Cell Signaling Technology) and mouse anti-HA 1:200 (Santa Cruz Biotechnology). Rat anti-Fz2 antibodies used at 1:300 were generated against the extracellular domain from residue 24 (start of the mature peptide) until residue 317 (just before the start of the 1st transmembrane domain), see supplementary Materials and methods for details. Fluorescent secondary antibodies used were Alexa-405, Alexa-488, Alexa-594 and Alexa-647 (Invitrogen) at 1:300 dilutions.

### Immunostaining, microscopy and image analysis

Extracellular Wg staining was performed as described previously (Strigini and Cohen, 2000). Total Wg staining was performed using conventional protocol also mentioned previously (Strigini and Cohen, 2000). The wing discs are fixed with 4% PFA and then permeabilized with 0.1% Triton X-100 in PBS, followed by antibody labeling. This protocol allows for the detection of both intracellular and extracellular Wg protein. Wing discs were mounted in Vectashield. Staining and microscopy conditions for discs used for comparisons were identical. Images were taken with a Leica SP5 or Olympus FV-3000 confocal and were analyzed using ImageJ software (Schneider et al., 2012). Plot analysis and intensity measurements were performed on raw data processed with ImageJ. Separate channel images were assembled using Adobe Photoshop CS5.1.

### Eclosion rate assay

To analyze eclosion rate 15 second-instar (∼60 h after egg laying) larvae of each genotype were separated from siblings using markers on the balancer chromosomes and grown in separate standard food vials at 25°C. Five independent sets with 15 larvae (total 75 larvae) were analyzed for each genotype. Number of flies eclosed for all the genotypes in five independent experiments were recorded. Eclosion percentage was calculated by measuring total number of animals eclosed for each genotype separately. Unpaired Student's *t*-test was used for statistical analysis.

### Heat-shock induction of ‘flip-out’ clones

**‘**Flip-out’ clones were generated by heat shocking mid-third-instar larvae for 30 min at 37°C. Larvae were then shifted to 18°C and were dissected 24 h later for antibody staining.

### *Drosophila* genotypes

The following genotypes were used in this study.

Fig. 1, Fig. S1: *y w hs-FLP/Actin-FRT-Stop-FRT-Gal4; UAS-Fz2/UAS-GFP*

Fig. S2A-D: *y w hs-FLP/Actin-FRT-CD2-Stop-FRT-Gal4; UAS-Fz2-ECD-GPI/ UAS-GFP*

Fig. S3: *y w hs-FLP/Actin-FRT-Stop-FRT-Gal4; NRT-wg/CyO-GFP; UAS-Fz2/ UAS-GFP*

Fig. 2A (left panel), Fig. 3A, Fig. S4A, Fig. 5A, Fig. S7A (graph): *w1118* (wild-type control)

Fig. 2A (right panel), Fig. 3B, Fig. S4B, Fig. 5B, Fig. S7A (graph): *NRT-wg/NRT-wg*

Fig. 3C, Fig. S4C,E: *ap-Gal4/+; UAS-GFP/UAS-wg-RNAi*

Fig. 3D, Fig. S4D,F: *ap-Gal4, NRT-wg/NRT-wg; UAS-GFP/UAS-wg-RNA*

Fig. 4A-E: *y w Ubx-FLP/+; wg-Gal4/UAS-evi-RNAi(KK); fz2^C1^, ri, FRT2A/ubi-GFP FRT2A*

Fig. S5A: *en-Gal4, UAS-GFP/UAS-evi-RNAi(KK)*

Fig. S5B,C: *y w Ubx-FLP/+; en-Gal4/UAS-evi-RNAi(KK); fz2^C1^, ri, FRT2A/ubi-GFP FRT2A*

Fig. S5D–F: *y w Ubx-FLP/+; +/+; fz2^C1^, ri, FRT2A/ubi-GFP FRT2A*

Fig. S6: *y w Ubx-FLP/+; +/+; fz^p21^FRT80/ubi-GFP FRT80*

Figs 6A, 7A: *dTomato-Dll* (control)

Figs 6B, 7B: *dTomato-Dll, UAS-fz2-RNAi/ +; hh-Gal4/ +*

Figs 6C, 7C: *RIV-FRT-NRT-wg-FRT-wg, dTomato-Dll / RIV-FRT-NRT-wg-FRT-wg*

Figs 6D, 7D, Fig. S7B: *RIV-FRT-NRT-wg-FRT-wg, dTomato-Dll, UAS-fz2-RNAi/ RIV-FRT-NRT-wg-FRT-wg; hh-GAL4/ +*

Fig. S7A (graph): *fz2^C1^, ri, FRT2A / Df(3L)fz2*

Fig. S7A (graph): *NRT-wg/NRT-wg; fz2^C1^, ri, FRT2A/Df(3L)fz2*

Fig. S8A,C: *dTomato-Dll, UAS-fz1-RNAi/ +; hh-Gal4/ +*

Fig. S8B,D: *RIV-FRT-NRT-wg-FRT-wg, dTomato-Dll, UAS-fz1-RNAi /RIV-FRT-NRT-wg-FRT-wg; hh-Gal4/ +*

## Supplementary Material

Supplementary information

## References

[DEV174789C1] Alexandre C., Baena-Lopez A. and Vincent J.-P. (2014). Patterning and growth control by membrane-tethered Wingless. *Nature* 505, 180-185. 10.1038/nature12879 24390349 PMC7611559

[DEV174789C2] Aulehla A., Wehrle C., Brand-Saberi B., Kemler R., Gossler A., Kanzler B. and Herrmann B. G. (2003). Wnt3a plays a major role in the segmentation clock controlling somitogenesis. *Dev. Cell* 4, 395-406. 10.1016/S1534-5807(03)00055-8 12636920

[DEV174789C3] Aulehla A., Wiegraebe W., Baubet V., Wahl M. B., Deng C., Taketo M., Lewandoski M. and Pourquié O. (2008). A beta-catenin gradient links the clock and wavefront systems in mouse embryo segmentation. *Nat. Cell Biol.* 10, 186-193. 10.1038/ncb1679 18157121 PMC7391962

[DEV174789C4] Baena-Lopez L. A., Alexandre C., Mitchell A., Pasakarnis L. and Vincent J. P. (2013). Accelerated homologous recombination and subsequent genome modification in Drosophila. *Development* 140, 4818-4825. 10.1242/dev.100933 24154526 PMC3833436

[DEV174789C5] Bänziger C., Soldini D., Schütt C., Zipperlen P., Hausmann G. and Basler K. (2006). Wntless, a conserved membrane protein dedicated to the secretion of Wnt proteins from signaling cells. *Cell* 125, 509-522. 10.1016/j.cell.2006.02.049 16678095

[DEV174789C6] Bartscherer K., Pelte N., Ingelfinger D. and Boutros M. (2006). Secretion of Wnt ligands requires Evi, a conserved transmembrane protein. *Cell* 125, 523-533. 10.1016/j.cell.2006.04.009 16678096

[DEV174789C7] Beaven R. and Denholm B. (2018). Release and spread of Wingless is required to pattern the proximo-distal axis of *Drosophila* renal tubules. *Elife* 7, e35373 10.7554/eLife.35373 30095068 PMC6086663

[DEV174789C8] Beckett K., Monier S., Palmer L., Alexandre C., Green H., Bonneil E., Raposo G., Thibault P., Le Borgne R. and Vincent J.-P. (2013). Drosophila S2 cells secrete wingless on exosome-like vesicles but the wingless gradient forms independently of exosomes. *Traffic* 14, 82-96. 10.1111/tra.12016 23035643 PMC4337976

[DEV174789C9] Ben-Zvi D., Pyrowolakis G., Barkai N. and Shilo B.-Z. (2011). Expansion-repression mechanism for scaling the Dpp activation gradient in Drosophila wing imaginal discs. *Curr. Biol.* 21, 1391-1396. 10.1016/j.cub.2011.07.015 21835621

[DEV174789C10] Bhanot P., Brink M., Samos C. H., Hsieh J.-C., Wang Y., Macke J. P., Andrew D., Nathans J. and Nusse R. (1996). A new member of the frizzled family from Drosophila functions as a Wingless receptor. *Nature* 382, 225-230. 10.1038/382225a0 8717036

[DEV174789C11] Bhanot P., Fish M., Jemison J. A., Nusse R., Nathans J. and Cadigan K. M. (1999). Frizzled and Dfrizzled-2 function as redundant receptors for Wingless during Drosophila embryonic development. *Development* 126, 4175-4186. 10457026 10.1242/dev.126.18.4175

[DEV174789C12] Bhat K. M. (1998). frizzled and frizzled 2 play a partially redundant role in wingless signaling and have similar requirements to wingless in neurogenesis. *Cell* 95, 1027-1036. 10.1016/S0092-8674(00)81726-2 9875856

[DEV174789C13] Boutros M. and Niehrs C. (2016). Sticking around: short-range activity of wnt ligands. *Dev. Cell* 36, 485-486. 10.1016/j.devcel.2016.02.018 26954543

[DEV174789C14] Cadigan K. M., Fish M. P., Rulifson E. J. and Nusse R. (1998). Wingless repression of Drosophila frizzled 2 expression shapes the Wingless morphogen gradient in the wing. *Cell* 93, 767-777. 10.1016/S0092-8674(00)81438-5 9630221

[DEV174789C15] Carron C., Pascal A., Djiane A., Boucaut J.-C., Shi D.-L. and Umbhauer M. (2003). Frizzled receptor dimerization is sufficient to activate the Wnt/beta-catenin pathway. *J. Cell Sci.* 116, 2541-2550. 10.1242/jcs.00451 12734397

[DEV174789C16] Chen C. M. and Struhl G. (1999). Wingless transduction by the Frizzled and Frizzled2 proteins of Drosophila. *Development* 126, 5441-5452. 10556068 10.1242/dev.126.23.5441

[DEV174789C17] Couso J. P., Bate M. and Martínez-Arias A. (1993). A wingless-dependent polar coordinate system in Drosophila imaginal discs. *Science* 259, 484-489. 10.1126/science.8424170 8424170

[DEV174789C18] Dekanty A. and Milán M. (2011). The interplay between morphogens and tissue growth. *EMBO Rep.* 12, 1003-1010. 10.1038/embor.2011.172 21886183 PMC3185346

[DEV174789C19] Estella C., McKay D. J. and Mann R. S. (2008). Molecular integration of wingless, decapentaplegic, and autoregulatory inputs into Distalless during Drosophila leg development. *Dev. Cell* 14, 86-96. 10.1016/j.devcel.2007.11.002 18194655 PMC2709787

[DEV174789C20] Farin H. F., Jordens I., Mosa M. H., Basak O., Korving J., Tauriello D. V. F., de Punder K., Angers S., Peters P. J., Maurice M. M. et al. (2016). Visualization of a short-range Wnt gradient in the intestinal stem-cell niche. *Nature* 530, 340-343. 10.1038/nature16937 26863187

[DEV174789C21] Galindo M. I., Fernández-Garza D., Phillips R. and Couso J. P. (2011). Control of Distal-less expression in the Drosophila appendages by functional 3′ enhancers. *Dev. Biol.* 353, 396-410. 10.1016/j.ydbio.2011.02.005 21320482 PMC3940868

[DEV174789C22] Gao B., Song H., Bishop K., Elliot G., Garrett L., English M. A., Andre P., Robinson J., Sood R., Minami Y. et al. (2011). Wnt signaling gradients establish planar cell polarity by inducing Vangl2 phosphorylation through Ror2. *Dev. Cell* 20, 163-176. 10.1016/j.devcel.2011.01.001 21316585 PMC3062198

[DEV174789C23] García-García M. J., Ramain P., Simpson P. and Modolell J. (1999). Different contributions of pannier and wingless to the patterning of the dorsal mesothorax of Drosophila. *Development* 126, 3523-3532. 10409499 10.1242/dev.126.16.3523

[DEV174789C24] Gavin B. J., McMahon J. A. and McMahon A. P. (1990). Expression of multiple novel Wnt-1/int-1-related genes during fetal and adult mouse development. *Genes Dev.* 4, 2319-2332. 10.1101/gad.4.12b.2319 2279700

[DEV174789C25] Gerlach J. P., Jordens I., Tauriello D. V. F., van ‘t Land-Kuper I., Bugter J. M., Noordstra I., van der Kooij J., Low T. Y., Pimentel-Muiños F. X., Xanthakis D. et al. (2018). TMEM59 potentiates Wnt signaling by promoting signalosome formation. *Proc. Natl. Acad. Sci. USA* 115, E3996-E4005. 10.1073/pnas.1721321115 29632210 PMC5924918

[DEV174789C26] Gierer A. and Meinhardt H. (1972). A theory of biological pattern formation. *Kybernetik* 12, 30-39. 10.1007/BF00289234 4663624

[DEV174789C27] Goodman R. M., Thombre S., Firtina Z., Gray D., Betts D., Roebuck J., Spana E. P. and Selva E. M. (2006). Sprinter: a novel transmembrane protein required for Wg secretion and signaling. *Development* 133, 4901-4911. 10.1242/dev.02674 17108000

[DEV174789C28] Greco V., Hannus M. and Eaton S. (2001). Argosomes: a potential vehicle for the spread of morphogens through epithelia. *Cell* 106, 633-645. 10.1016/S0092-8674(01)00484-6 11551510

[DEV174789C29] Gross J. C., Chaudhary V., Bartscherer K. and Boutros M. (2012). Active Wnt proteins are secreted on exosomes. *Nat. Cell Biol.* 14, 1036-1045. 10.1038/ncb2574 22983114

[DEV174789C30] Huang H. and Kornberg T. B. (2015). Myoblast cytonemes mediate Wg signaling from the wing imaginal disc and Delta-Notch signaling to the air sac primordium. *Elife* 4, e06114 10.7554/eLife.06114 25951303 PMC4423120

[DEV174789C31] Jones K. H., Liu J. and Adler P. N. (1996). Molecular analysis of EMS-induced frizzled mutations in Drosophila melanogaster. *Genetics* 142, 205-215. 8770598 10.1093/genetics/142.1.205 PMC1206949

[DEV174789C32] Kennerdell J. R. and Carthew R. W. (1998). Use of dsRNA-mediated genetic interference to demonstrate that frizzled and frizzled 2 act in the wingless pathway. *Cell* 95, 1017-1026. 10.1016/S0092-8674(00)81725-0 9875855

[DEV174789C33] Kiecker C. and Niehrs C. (2001). A morphogen gradient of Wnt/beta-catenin signalling regulates anteroposterior neural patterning in Xenopus. *Development* 128, 4189-4201. 11684656 10.1242/dev.128.21.4189

[DEV174789C34] Korkut C., Ataman B., Ramachandran P., Ashley J., Barria R., Gherbesi N. and Budnik V. (2009). Trans-synaptic transmission of vesicular Wnt signals through Evi/Wntless. *Cell* 139, 393-404. 10.1016/j.cell.2009.07.051 19837038 PMC2785045

[DEV174789C35] Luga V., Zhang L., Viloria-Petit A. M., Ogunjimi A. A., Inanlou M. R., Chiu E., Buchanan M., Hosein A. N., Basik M. and Wrana J. L. (2012). Exosomes mediate stromal mobilization of autocrine Wnt-PCP signaling in breast cancer cell migration. *Cell* 151, 1542-1556. 10.1016/j.cell.2012.11.024 23260141

[DEV174789C36] Mattes B., Dang Y., Greicius G., Kaufmann L. T., Prunsche B., Rosenbauer J., Stegmaier J., Mikut R., Özbek S., Nienhaus G. U. et al. (2018). Wnt/PCP controls spreading of Wnt/β-catenin signals by cytonemes in vertebrates. *Elife* 7, e36953 10.7554/eLife.36953 30060804 PMC6086664

[DEV174789C37] Montagne J., Stewart M. J., Stocker H., Hafen E., Kozma S. C. and Thomas G. (1999). Drosophila S6 kinase: a regulator of cell size. *Science* 285, 2126-2129. 10.1126/science.285.5436.2126 10497130

[DEV174789C38] Morata G. and Struhl G. (2014). Developmental biology: tethered wings. *Nature* 505, 162-163. 10.1038/nature12848 24390347

[DEV174789C39] Müller H. A., Samanta R. and Wieschaus E. (1999). Wingless signaling in the Drosophila embryo: zygotic requirements and the role of the frizzled genes. *Development* 126, 577-586. 9876186 10.1242/dev.126.3.577

[DEV174789C40] Mulligan K. A., Fuerer C., Ching W., Fish M., Willert K. and Nusse R. (2012). Secreted Wingless-interacting molecule (Swim) promotes long-range signaling by maintaining Wingless solubility. *Proc. Natl. Acad. Sci. USA* 109, 370-377. 10.1073/pnas.1119197109 22203956 PMC3258625

[DEV174789C41] Neumann C. J. and Cohen S. M. (1997). Long-range action of Wingless organizes the dorsal-ventral axis of the Drosophila wing. *Development* 124, 871-880. 9043068 10.1242/dev.124.4.871

[DEV174789C42] Neumann S., Coudreuse D. Y. M., van der Westhuyzen D. R., Eckhardt E. R. M., Korswagen H. C., Schmitz G. and Sprong H. (2009). Mammalian Wnt3a is released on lipoprotein particles. *Traffic* 10, 334-343. 10.1111/j.1600-0854.2008.00872.x 19207483

[DEV174789C43] Ng M., Diaz-Benjumea F. J., Vincent J.-P., Wu J. and Cohen S. M. (1996). Specification of the wing by localized expression of wingless protein. *Nature* 381, 316-318. 10.1038/381316a0 8692268

[DEV174789C44] Niehrs C. (2010). On growth and form: a Cartesian coordinate system of Wnt and BMP signaling specifies bilaterian body axes. *Development* 137, 845-857. 10.1242/dev.039651 20179091

[DEV174789C45] Nusse R. and Clevers H. (2017). Wnt/β-Catenin signaling, disease, and emerging therapeutic modalities. *Cell* 169, 985-999. 10.1016/j.cell.2017.05.016 28575679

[DEV174789C46] Panáková D., Sprong H., Marois E., Thiele C. and Eaton S. (2005). Lipoprotein particles are required for Hedgehog and Wingless signalling. *Nature* 435, 58-65. 10.1038/nature03504 15875013

[DEV174789C47] Pani A. M. and Goldstein B. (2018). Direct visualization of a native Wnt. *Elife* 7, e38325 10.7554/eLife.38325 30106379 PMC6143344

[DEV174789C48] Pérez L., Barrio L., Cano D., Fiuza U.-M., Muzzopappa M. and Milán M. (2011). Enhancer-PRE communication contributes to the expansion of gene expression domains in proliferating primordia. *Development* 138, 3125-3134. 10.1242/dev.065599 21715425

[DEV174789C49] Pfeiffer S., Alexandre C., Calleja M. and Vincent J. P. (2000). The progeny of wingless-expressing cells deliver the signal at a distance in Drosophila embryos. *Curr. Biol.* 10, 321-324. 10.1016/S0960-9822(00)00381-X 10744976

[DEV174789C50] Piddini E. and Vincent J.-P. (2009). Interpretation of the wingless gradient requires signaling-induced self-inhibition. *Cell* 136, 296-307. 10.1016/j.cell.2008.11.036 19167331

[DEV174789C51] Piddini E., Marshall F., Dubois L., Hirst E. and Vincent J. P. (2005). Arrow (LRP6) and Frizzled2 cooperate to degrade Wingless in Drosophila imaginal discs. *Development* 132, 5479-5489. 10.1242/dev.02145 16291792

[DEV174789C52] Port F. and Bullock S. L. (2016). Augmenting CRISPR applications in Drosophila with tRNA-flanked sgRNAs. *Nat. Methods* 13, 852-854. 10.1038/nmeth.3972 27595403 PMC5215823

[DEV174789C53] Port F., Chen H.-M., Lee T. and Bullock S. L. (2014). Optimized CRISPR/Cas tools for efficient germline and somatic genome engineering in Drosophila. *Proc. Natl. Acad. Sci. USA* 111, E2967-E2976. 10.1073/pnas.1405500111 25002478 PMC4115528

[DEV174789C54] Rogers K. W. and Schier A. F. (2011). Morphogen gradients: from generation to interpretation. *Annu. Rev. Cell Dev. Biol.* 27, 377-407. 10.1146/annurev-cellbio-092910-154148 21801015

[DEV174789C55] Sato A., Kojima T., Ui-Tei K., Miyata Y. and Saigo K. (1999). Dfrizzled-3, a new Drosophila Wnt receptor, acting as an attenuator of Wingless signaling in wingless hypomorphic mutants. *Development* 126, 4421-4430. 10498678 10.1242/dev.126.20.4421

[DEV174789C56] Schneider C. A., Rasband W. S. and Eliceiri K. W. (2012). NIH Image to ImageJ: 25 years of image analysis. *Nat. Methods* 9, 671-675. 10.1038/nmeth.2089 22930834 PMC5554542

[DEV174789C58] Stanganello E. and Scholpp S. (2016). Role of cytonemes in Wnt transport. *J. Cell Sci.* 129, 665-672. 10.1242/jcs.182469 26823607

[DEV174789C59] Stanganello E., Hagemann A. I. H., Mattes B., Sinner C., Meyen D., Weber S., Schug A., Raz E. and Scholpp S. (2015). Filopodia-based Wnt transport during vertebrate tissue patterning. *Nat. Commun.* 6, 5846 10.1038/ncomms6846 25556612

[DEV174789C72] Stewart R. A., Ramakrishnan A.-B. and Cadigan K. M. (2019). Diffusion and function of Wnt ligands. *PLoS Genet.* 15, e1008154 10.1371/journal.pgen.1008154 31194739 PMC6563952

[DEV174789C60] Strigini M. and Cohen S. M. (2000). Wingless gradient formation in the Drosophila wing. *Curr. Biol.* 10, 293-300. 10.1016/S0960-9822(00)00378-X 10744972

[DEV174789C61] Struhl G. and Basler K. (1993). Organizing activity of wingless protein in Drosophila. *Cell* 72, 527-540. 10.1016/0092-8674(93)90072-X 8440019

[DEV174789C62] Tanimoto H., Itoh S., ten Dijke P. and Tabata T. (2000). Hedgehog creates a gradient of DPP activity in Drosophila wing imaginal discs. *Mol. Cell* 5, 59-71. 10.1016/S1097-2765(00)80403-7 10678169

[DEV174789C63] Thompson B. J. and Cohen S. M. (2006). The Hippo pathway regulates the bantam microRNA to control cell proliferation and apoptosis in Drosophila. *Cell* 126, 767-774. 10.1016/j.cell.2006.07.013 16923395

[DEV174789C71] Tian A., Duwadi D., Benchabane H. and Ahmed Y. (2019). Essential long-range action of Wingless/Wnt in adult intestinal compartmentalization. *PLoS Genet* 15, e1008111 10.1371/journal.pgen.1008111 31194729 PMC6563961

[DEV174789C64] Vuilleumier R., Springhorn A., Patterson L., Koidl S., Hammerschmidt M., Affolter M. and Pyrowolakis G. (2010). Control of Dpp morphogen signalling by a secreted feedback regulator. *Nat. Cell Biol.* 12, 611-617. 10.1038/ncb2064 20453847

[DEV174789C65] Wehrli M., Dougan S. T., Caldwell K., O'Keefe L., Schwartz S., Vaizel-Ohayon D., Schejter E., Tomlinson A. and DiNardo S. (2000). arrow encodes an LDL-receptor-related protein essential for Wingless signalling. *Nature* 407, 527-530. 10.1038/35035110 11029006

[DEV174789C66] Williams J. A., Paddock S. W. and Carroll S. B. (1993). Pattern formation in a secondary field: a hierarchy of regulatory genes subdivides the developing Drosophila wing disc into discrete subregions. *Development* 117, 571-584. 8330528 10.1242/dev.117.2.571

[DEV174789C67] Wolpert L. (1969). Positional information and the spatial pattern of cellular differentiation. *J. Theor. Biol.* 25, 1-47. 10.1016/S0022-5193(69)80016-0 4390734

[DEV174789C68] Wolpert L. (1971). Positional information and pattern formation. *Curr. Top. Dev. Biol.* 6, 183-224. 10.1016/S0070-2153(08)60641-9 4950136

[DEV174789C69] Zecca M., Basler K. and Struhl G. (1996). Direct and long-range action of a wingless morphogen gradient. *Cell* 87, 833-844. 10.1016/S0092-8674(00)81991-1 8945511

[DEV174789C70] Zhan T., Rindtorff N. and Boutros M. (2017). Wnt signaling in cancer. *Oncogene* 36, 1461-1473. 10.1038/onc.2016.304 27617575 PMC5357762

