# Supplementary material for: Robust Wnt signaling is maintained by a Wg protein gradient and Fz2 receptor activity in the developing Drosophila wing
Source: Development. 2019 Aug 9;146(15):dev174789. doi: 10.1242/dev.174789 (PMC6703709; doi:10.1242/dev.174789)
Supplement: Supplementary information [file develop-146-174789-s1.pdf]

SUPPLEMENTAL FIGURES

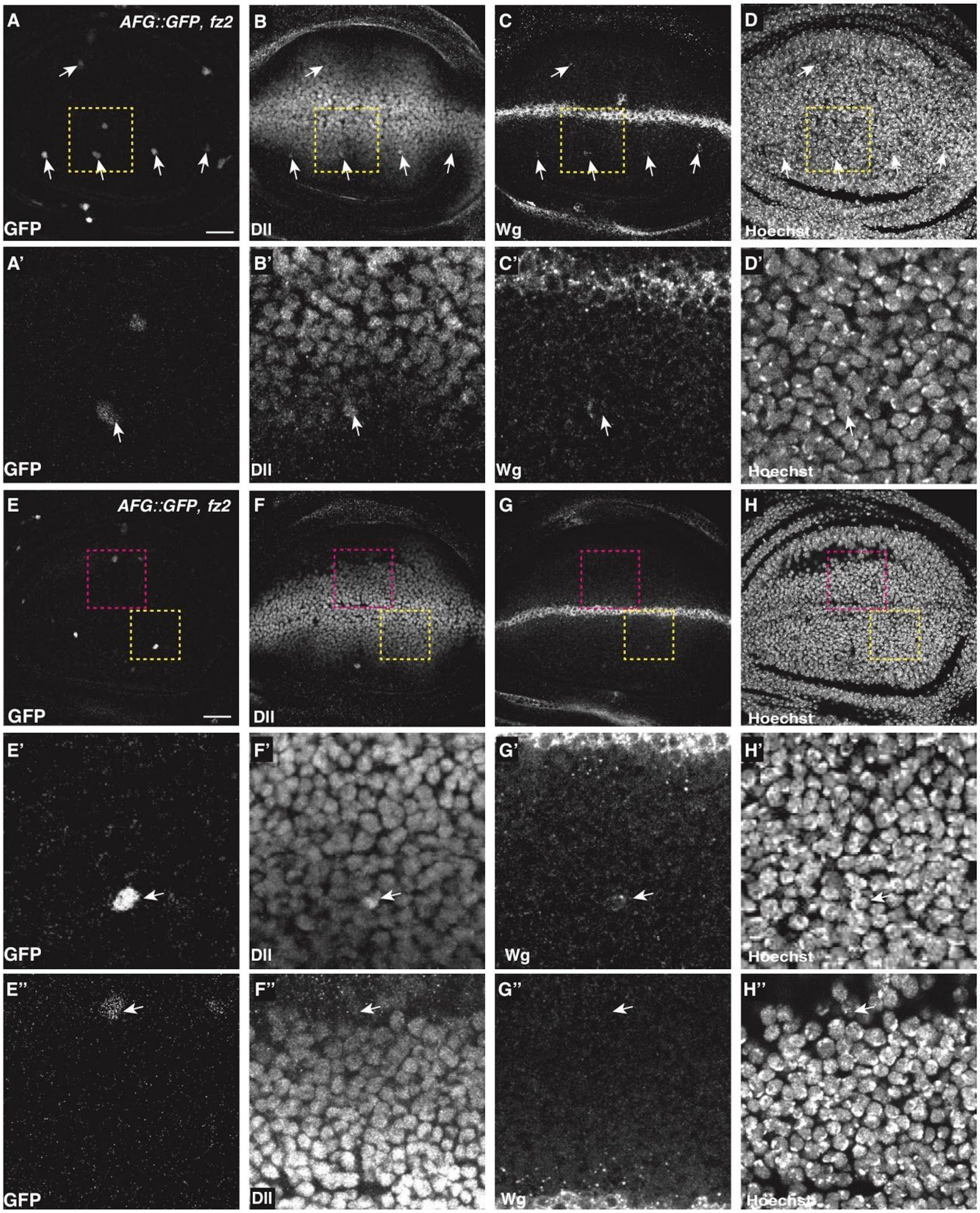

Figure S1: Single-cell clones expressing Fz2 show Wg accumulation and enhanced signaling. To supplement Figure 1, panels provide further two examples (A-D and E-H) of Wg accumulation and Dll upregulation in single-cell clones overexpressing Fz2. A'-D' show magnified images of the area marked by yellow box in A-D. E'-H' show magnified images of the area marked by yellow box in E-H. E''-H'' show magnified images of the area marked by red box in E-H. Scale bar 20 μm.

Development • Supplementary information

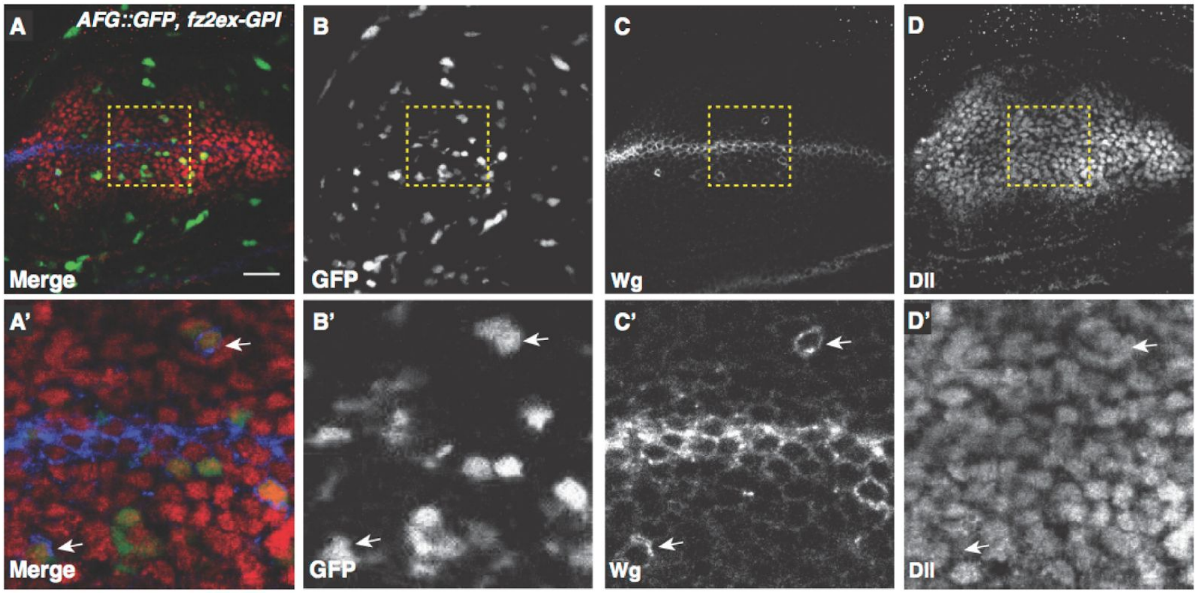

Figure S2: Formation of functional Wg-Fz2 complexes is required for activation of Wg signaling at a distance.

(A–D) Flip-out clones overexpressing dominant-negative Fz2 (marked with GFP). (A'–D') Area marked with yellow box in A–D. 24 hours after the heat-shock to induce DN-Fz2 expressing single-cell clones, total Wg staining shows clones with accumulation of Wg (C and C' arrows), however Dll levels were unaffected (D, D' arrows). Images show projection of 3 confocal slices. Scale bar 20 μm.

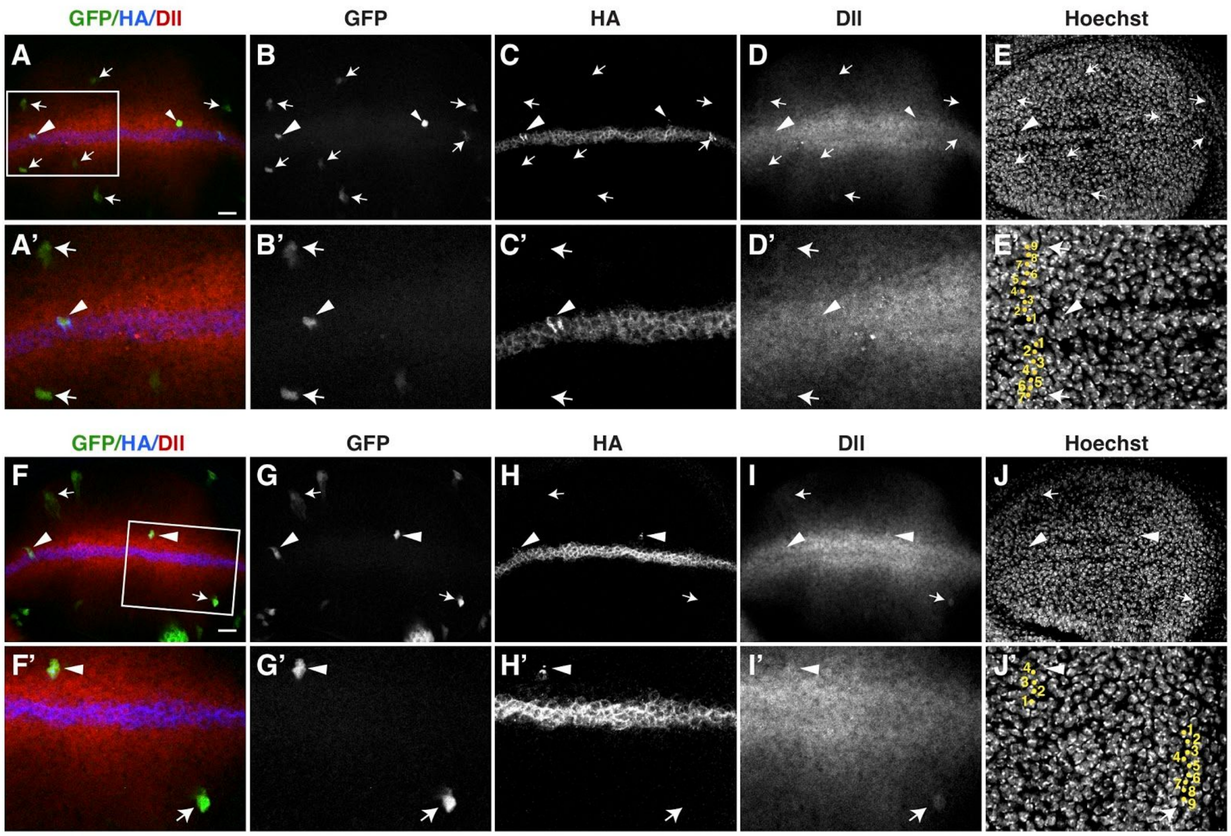

Figure S3: Accumulation of HA- tagged NRT-Wg in Fz2 overexpressing single cell clones.
(A-J) Images represent two samples of the wing discs (sample1 shown in A-E and sample2 shown in F-J) containing single cell flip-out clones overexpressing Fz2 in *NRT-wg/Cyo-GFP* heterozygous background (marked by GFP, arrows and arrowheads). NRT-Wg levels (measured by HA staining) were increased mostly in clones close to Wg producing cells (arrowheads in A, C and F, H). The white boxes marked in A and F were enlarged and merge for 3 confocal slices was used to measure the distance of Fz2 overexpressing clones from Wg producing cells by marking the nuclei with yellow dots in both wing discs samples (E' and J'). HA-positive NRT-Wg can be detected in clone at 4 cell distances from Wg producing cells (arrowheads H' and J'). Clones present at a either 7 or 9 cell distances did not show accumulation of NRT-Wg (arrows C', E' and H' and J'), however Dll expression (measured using endogenously Tdtomato tagged Dll) was mildly regulated in these clones (arrows D' and I'). Scale Bar 20 μm. (Total N=4).

Development • Supplementary information

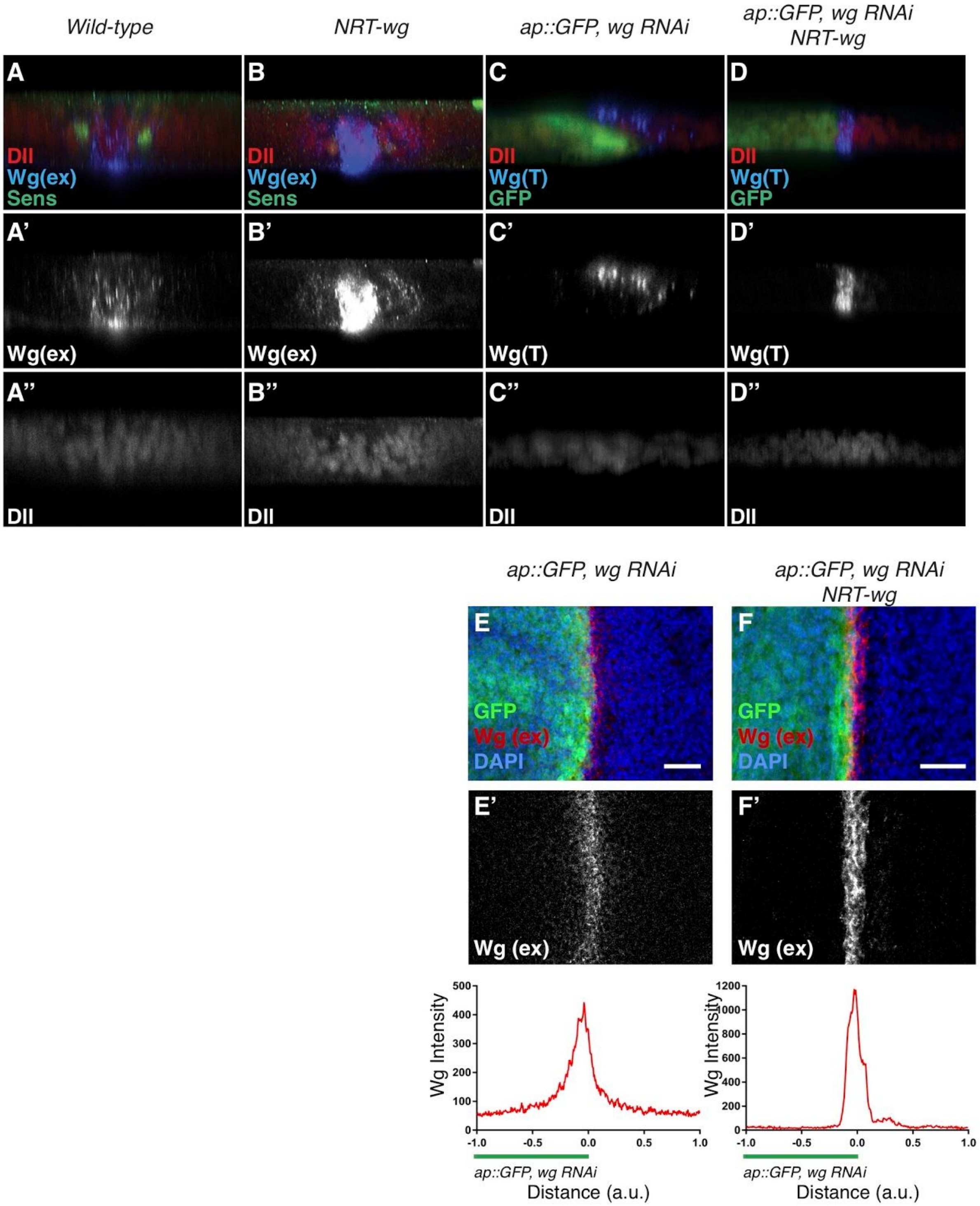

Figure S4: Secreted Wg regulates target gene expression across the DV compartment boundary. (A-B) Cross-section view of extracellular Wg staining and Dll staining performed on either wild-type discs (A–A'') or homozygous *NRT-wg* discs and imaged with same confocal settings (B–B''). (C–D) Cross-section view of total Wg and Dll staining on discs with depletion of *wg* in dorsal compartment with *ap-Gal4* in either wild-type background (C–C'') or homozygous *NRT-wg* background (D–D''). Extracellular Wg staining on discs with depletion of *wg* with *ap-Gal4* in either wild-type background (E–E') or homozygous *NRT-wg* background (F–F''). a.u.= arbitrary unit. Scale bar 20 µm.

Development • Supplementary information

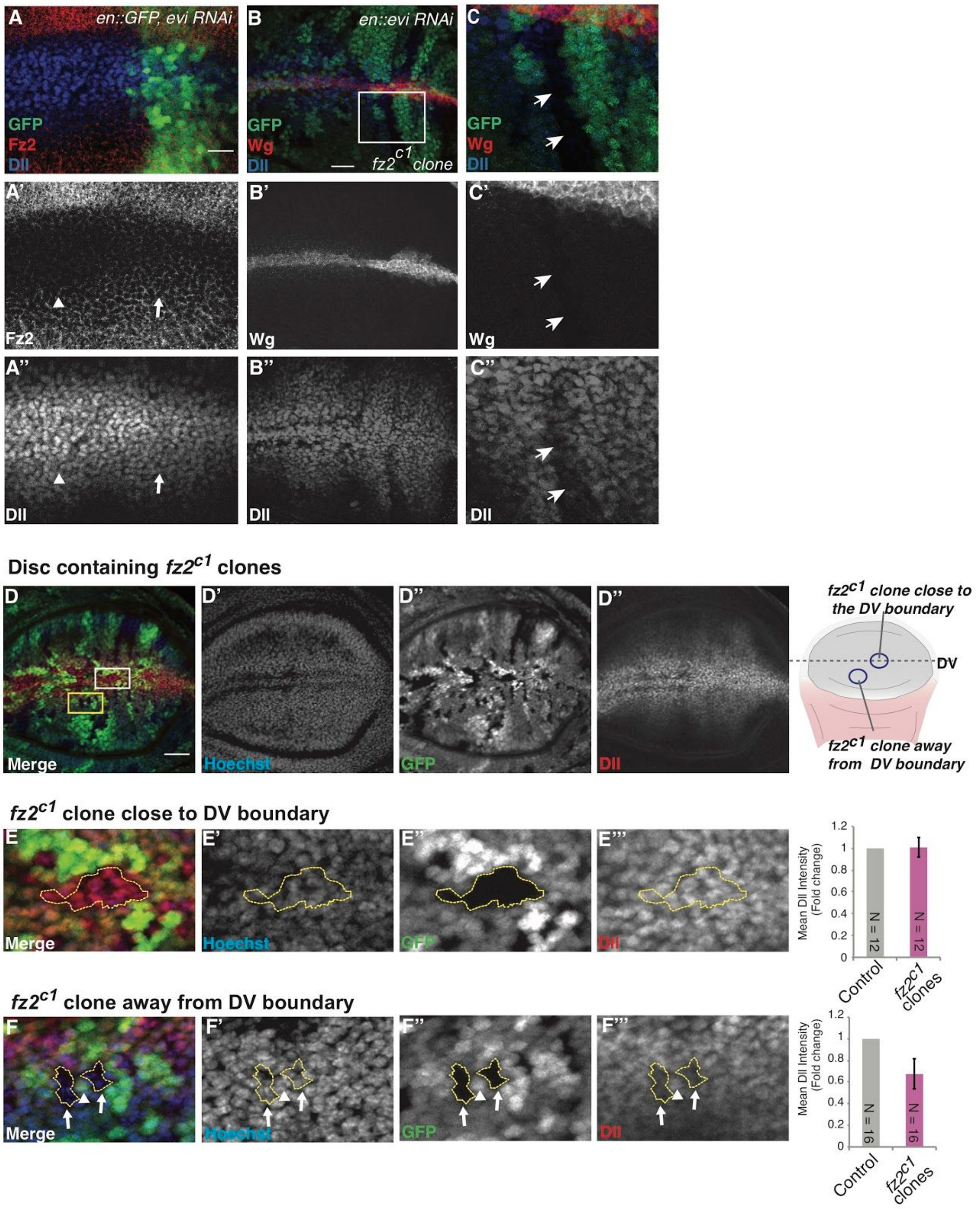

Figure S5: Fz2 is required for the maintenance of long-range Wg target gene expression.
(A–A'') Depletion of Evi in the posterior compartment of the disc shows up-regulation of Fz2 (compare arrowhead to arrow in A') while Dll levels are similar (compare arrowhead to arrow in A''). (B–B'') Depletion of Evi in the posterior compartment of the disc (identified with Wg accumulation) in the background of *fz2^{c1}* clones (marked by absence of GFP) show reduced levels of Dll inside the mutant clones. (C–C'') Enlarged images of *fz2* mutant clone (marked by white box in B). (D–F) *fz2^{c1}* clones in otherwise wild-type disc show no effect on Dll levels in clones close to the DV boundary (marked by white box in D). (E–E''') Enlarged

Development • Supplementary information

images of the *fz2*^{c1} clones found close to the DV boundary. (F'-F'') Enlarged images of *fz2*^{c1} found further away from the DV boundary (marked by yellow box in D) show reduced Dll levels inside the clones (F''' arrows) compared to control cells at the same distance (F''' arrowhead). Quantification shows Dll intensity measurement inside the clone and Dll intensity of the same area from neighbouring control cells. N (marked on the graphs) represents combined number of clones from several discs. Scale bar 20 µm.

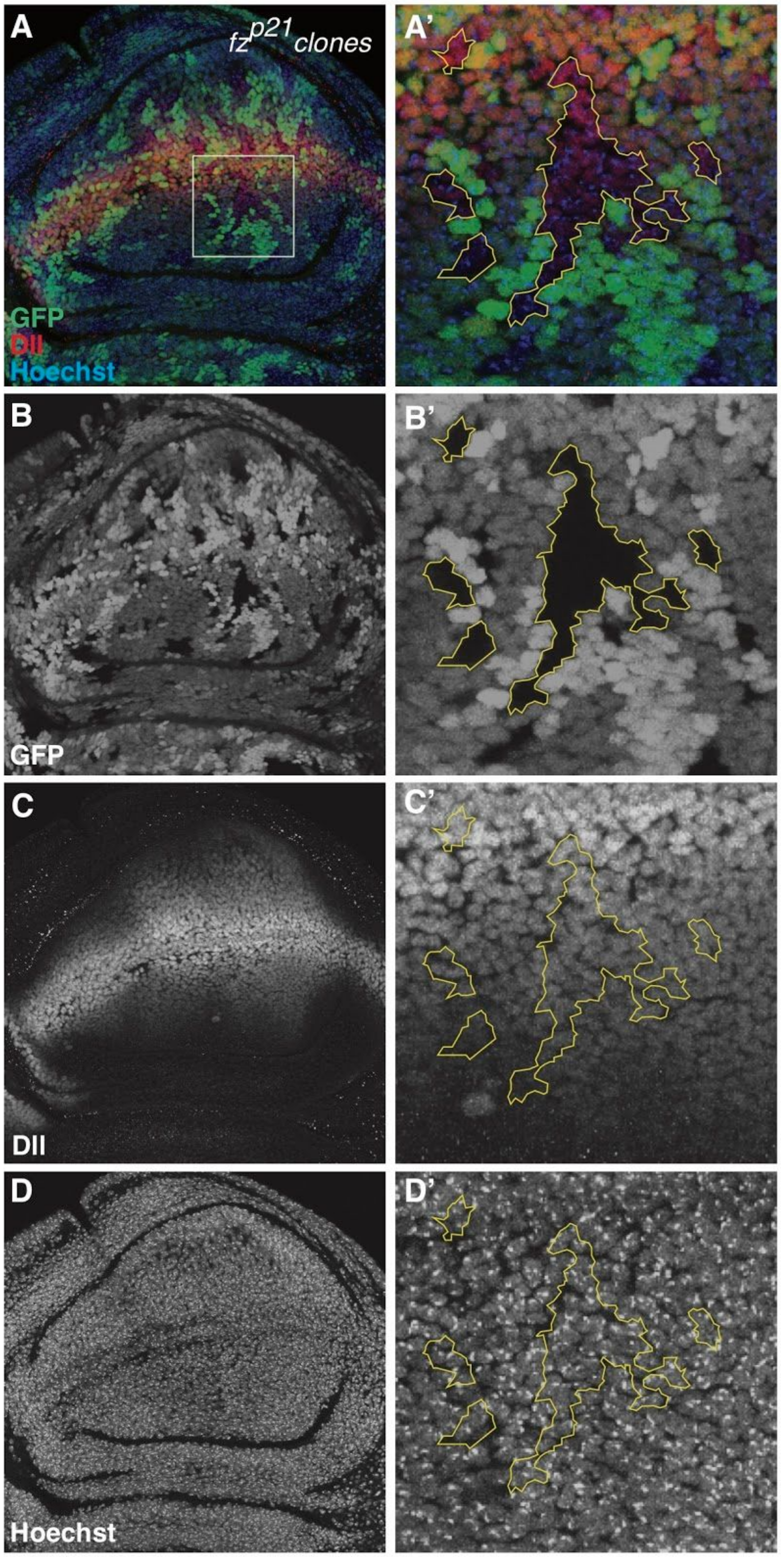

Figure S6: Fz1 is not required for the maintenance of long-range Wg target gene expression, (A–D) *fz*^{p21} clones in otherwise wild-type disc show no effect on Dll levels in clones. (A'–D') Enlarged images of the *fz*^{p21} clones (marked by white box in A) show no significant difference in the Dll expression even at a distance from DV boundary. Scale bar 20 µm.

Development • Supplementary information

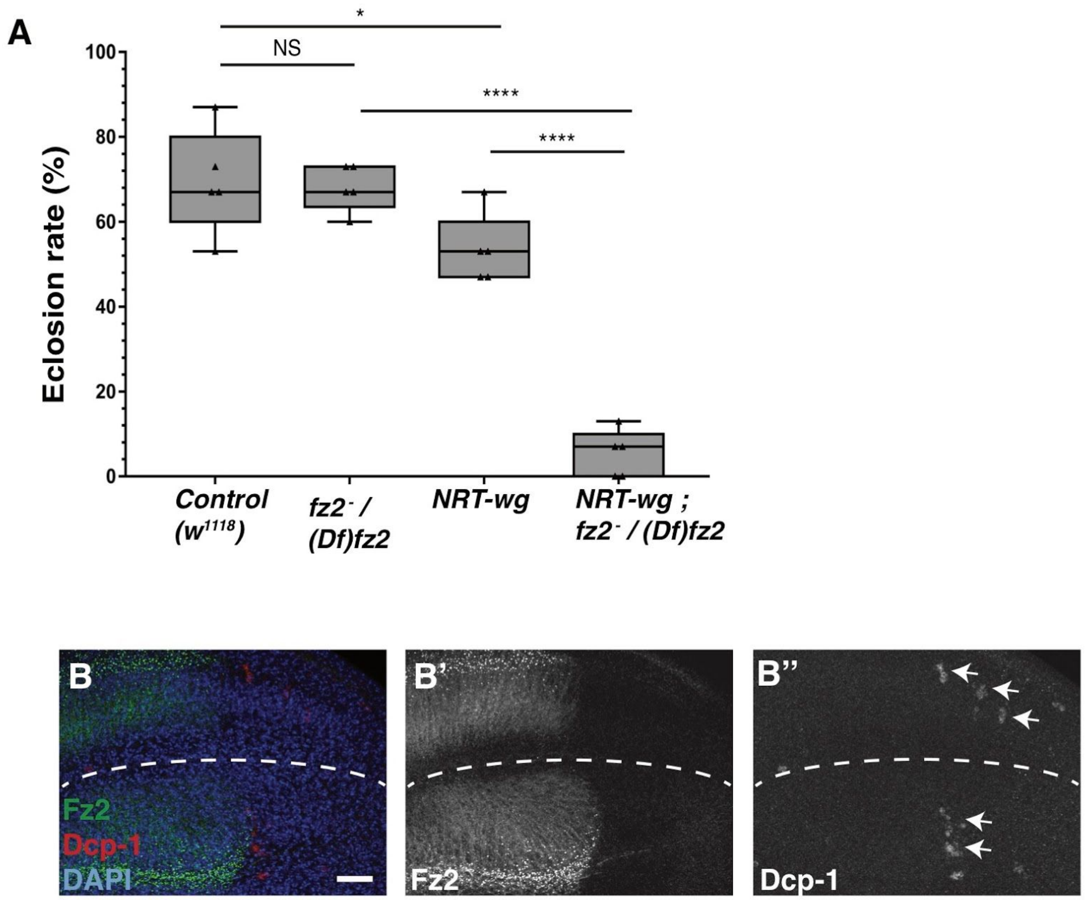

Figure S7: *Removal of Fz2* reduces survival of *NRT-wg* flies and induces cell death in *NRT-wg* wing imaginal discs. .

A) The box plot graph depicts eclosion rate percentage of control (w^{1118}), trans-heterozygous *fz2* LOF (*$fz2^{C1}$, ri, FRT2A / Df(3L)fz2),* homozygous *NRT-wg* and trans-heterozygous *fz2* LOF in *NRT-wg* (*NRT-wg / NRT-wg ; $fz2^{C1}$, ri, FRT2A / Df(3L)fz2*) animals cultured separately without siblings. *NRT-wg* homozygous show reduction in survival compared to control and trans-heterozygous *fz2* LOF flies (p=0.0415), while trans-heterozygous *fz2* LOF in the *NRT-wg* flies have significant reduction in the survival rate (p<0.0001). 75 animals were used in 5 experiments performed for each genotype. Statistical significance was assessed using unpaired Student's t-test. **B**) Fz2 and Dcp-1 expression upon *fz2* knockdown in the posterior compartment of *NRT-wg* discs. Dotted white line represent DV boundary and arrows marked Dcp-1 expressing cells (also see Fig. 7D). Scale bar 20 µm.

Development • Supplementary information

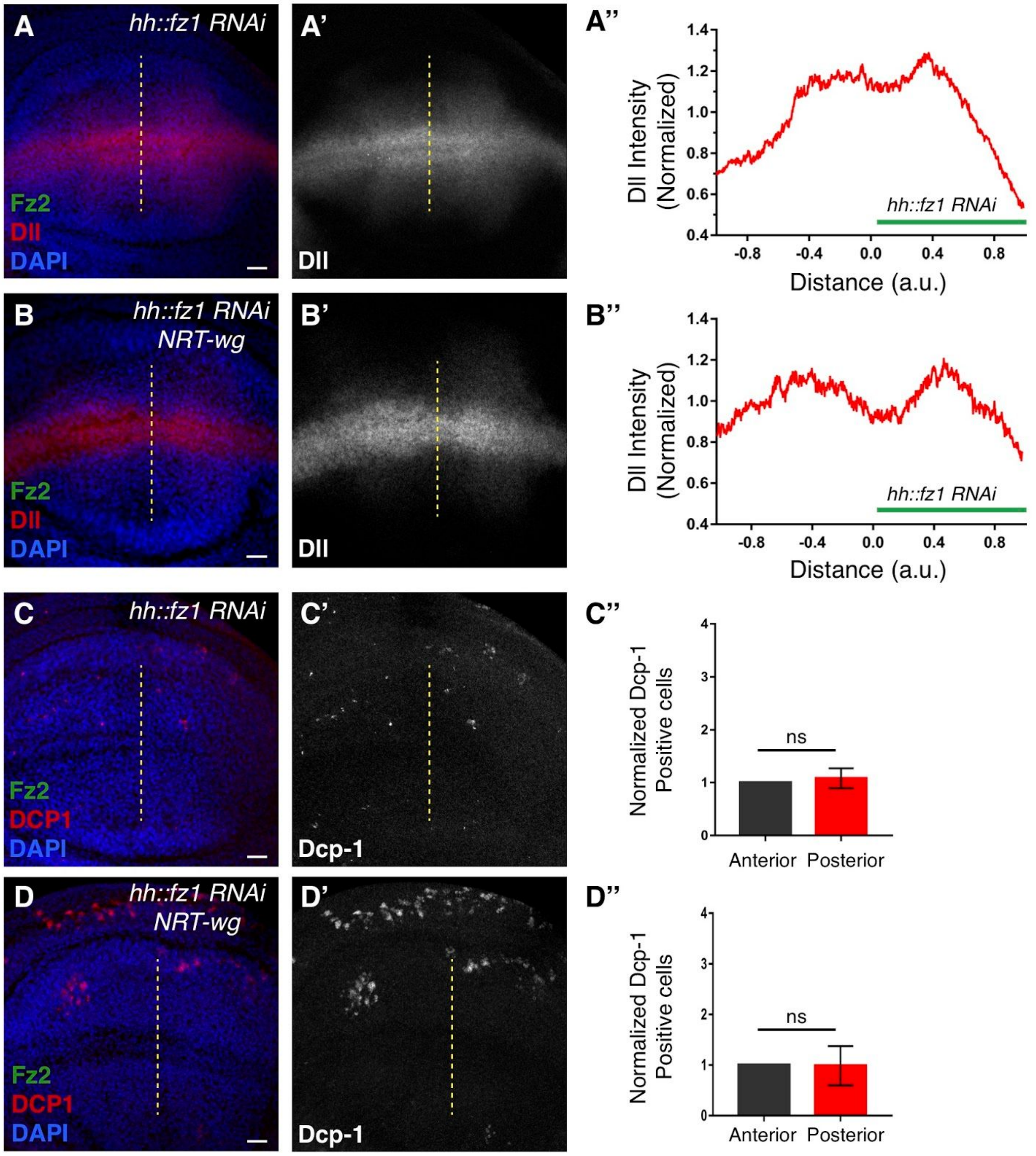

Figure S8: Dll levels and cell death are unaffected upon loss of Fz1 in *NRT-wg* flies.

Dll expression (measured using endogenously dTomato Dll reporter) in unaffected upon *fz1* knockdown in posterior compartment of wing imaginal disc with *hh-Gal4* in both *wild-type* (N=4) (A-A') and *NRT-wg* (N=5) (B-B') background. (A'' and B'') The graph depicts the Dll intensity profile across the red box for the respective genotypes. Dcp-1 levels are not changed upon Fz1 knockdown in posterior compartment of wing imaginal disc, both in wild-type (C-C') and *NRT-wg* (D-D') background. (C''-D'') The graph represents normalized Dcp-1 positive cells in the posterior compartment compared to the anterior compartment for the respective genotypes. Unpaired Student's t-test was applied for statistical analysis. a.u.= arbitrary unit. Scale bar 20 µm.

Supplemental Materials and Methods

Fz2 antibody generation

Fz2 antibodies were generated by amplifying the N-term region of the *fz2* gene using the following primers; forward primer (5'-CGGAATTCCATATGGATGGACCGCTGCACAGTGCG-3') and reverse primer (5'-GCGCAGATCTTTACGCGAAATCCTTTTCGTCGTT-3'). The PCR product obtained was cloned into pET vector (vector used for the expression of proteins in bacteria). pET Fz2-N-term fusion protein construct was transformed into BL21 (DE3) pLysS cells Rosetta strain (Novagen) which were grown overnight in Ampicillin sucrose agar plates at 37ºC. Next day, colonies were resuspended in 2 ml of culture medium, which was then inoculated into 400ml Terrific Broth medium in a baffle flask (360 ml TB + 40 ml K-PO4 + 100 ug/ml Carbenicillin). This was further grown at 37ºC/110 rpm until the OD [600] = 1.0. Culture was then induced with Isopropyl β-D-1-thiogalactopyranoside (IPTG) with 1mM final concentration, after which it was further grown for 3-4 hrs at 37ºC and the induced protein was harvested under denaturing conditions. Purification of the His tag fusion protein was done using His-Bind resin (Novagen) following the standard protocol, finally eluted in 1X Elution buffer/8M Urea (1M imidazole, 0.5M NaCl, 20mM Tris pH7.9, 8M Urea). Urea was then removed by dialysis against lowering concentration of Urea in PBS at 4ºC. Fusion protein was finally stored in PBS and was immunized in rats (University of Sheffield immunization facility).

Development • Supplementary information
